# Screening for lung cancer with computed tomography: protocol for systematic reviews for the Canadian Task Force on Preventive Health Care

**DOI:** 10.1186/s13643-024-02506-3

**Published:** 2024-03-16

**Authors:** Jennifer Pillay, Sholeh Rahman, Scott Klarenbach, Donna L. Reynolds, Laure A. Tessier, Guylène Thériault, Nav Persaud, Christian Finley, Natasha Leighl, Matthew D. F. McInnes, Chantelle Garritty, Gregory Traversy, Maria Tan, Lisa Hartling

**Affiliations:** 1 Alberta Research Centre for Health Evidence, University of Alberta, 11405 87 Avenue NW, Edmonton, Alberta T6G 1C9 Canada; 2 Department of Medicine, University of Alberta, Edmonton, Canada; 3 Dalla Lana School of Public Health and Department of Family and Community Medicine, University of Toronto, Toronto, Canada; 4 Global Health and Guidelines Division, Centre for Chronic Disease Prevention and Health Equity, Public Health Agency of Canada, Ottawa, Canada; 5 Department of Family Medicine, McGill University, Montreal, Canada; 6 Department of Family Medicine, University of Toronto Faculty of Medicine, Toronto, Canada; 7 Department of Surgery (Division of Thoracic Surgery), McMaster University, Hamilton, Canada; 8 Department of Medicine, University of Toronto, Toronto, Canada; 9 Departments of Radiology and Epidemiology, University of Ottawa, Ottawa, Canada

**Keywords:** Systematic review, Guideline, Lung cancer, Mass screening

## Abstract

**Purpose:**

Lung cancer is the leading cause of cancer deaths in Canada, and because early cancers are often asymptomatic screening aims to prevent mortality by detecting cancer earlier when treatment is more likely to be curative. These reviews will inform updated recommendations by the Canadian Task Force on Preventive Health Care on screening for lung cancer.

**Methods:**

We will update the review on the benefits and harms of screening with CT conducted for the task force in 2015 and perform de novo reviews on the comparative effects between (i) trial-based selection criteria and use of risk prediction models and (ii) trial-based nodule classification and different nodule classification systems and on patients’ values and preferences. We will search Medline, Embase, and Cochrane Central (for questions on benefits and harms from 2015; comparative effects from 2012) and Medline, Scopus, and EconLit (for values and preferences from 2012) via peer-reviewed search strategies, clinical trial registries, and the reference lists of included studies and reviews. Two reviewers will screen all citations (including those in the previous review) and base inclusion decisions on consensus or arbitration by another reviewer. For benefits (i.e., all-cause and cancer-specific mortality and health-related quality of life) and harms (i.e., overdiagnosis, false positives, incidental findings, psychosocial harms from screening, and major complications and mortality from invasive procedures as a result of screening), we will include studies of adults in whom lung cancer is not suspected. We will include randomized controlled trials comparing CT screening with no screening or alternative screening modalities (e.g., chest radiography) or strategies (e.g., CT using different screening intervals, classification systems, and/or patient selection via risk models or biomarkers); non-randomized studies, including modeling studies, will be included for the comparative effects between trial-based and other selection criteria or nodule classification methods. For harms (except overdiagnosis) we will also include non-randomized and uncontrolled studies. For values and preferences, the study design may be any quantitative design that either directly or indirectly measures outcome preferences on outcomes pertaining to lung cancer screening. We will only include studies conducted in Very High Human Development Countries and having full texts in English or French. Data will be extracted by one reviewer with verification by another, with the exception of result data on mortality and cancer incidence (for calculating overdiagnosis) where duplicate extraction will occur. If two or more studies report on the same comparison and it is deemed suitable, we will pool continuous data using a mean difference or standardized mean difference, as applicable, and binary data using relative risks and a DerSimonian and Laird model unless events are rare (< 1%) where we will pool odds ratios using Peto’s method or (if zero events) the reciprocal of the opposite treatment arm size correction. For pooling proportions, we will apply suitable transformation (logit or arcsine) depending on the proportions of events. If meta-analysis is not undertaken we will synthesize the data descriptively, considering clinical and methodological differences. For each outcome, two reviewers will independently assess within- and across-study risk of bias and rate the certainty of the evidence using GRADE (Grading of Recommendations Assessment, Development, and Evaluation), and reach consensus.

**Discussion:**

Since 2015, additional trials and longer follow-ups or additional data (e.g., harms, specific patient populations) from previously published trials have been published that will improve our understanding of the benefits and harms of screening. The systematic review of values and preferences will allow fulsome insights that will inform the balance of benefits and harms.

**Systematic review registration:**

PROSPERO CRD42022378858

**Supplementary Information:**

The online version contains supplementary material available at 10.1186/s13643-024-02506-3.

## Background

### Description of condition

Lung cancer is divided into two main types: small-cell lung cancer (SCLC) and non-small-cell lung cancer (NSCLC). SCLC is diagnosed much less frequently than NSCLC; the latter accounts for about 9 in 10 lung cancer cases [1]. NSCLC is further classified into histologic subtypes, the most common being adenocarcinoma, squamous cell carcinoma, and large cell carcinoma. The tumor node metastasis staging system characterizes the extent of the disease and determines the lung cancer stage, treatment, and prognosis. Major stage groupings are stage 0 (carcinoma in situ; nonmalignant) through IV (malignancy spread to the other lung or outside chest). Stage I disease involves tumors ≤ 4 cm, and no lymph node or metastatic involvement, while stage IV cancers have one or more distant metastases [2].

### Incidence and burden of disease

Although rates have been declining in the past two decades, lung cancer was projected for 2023 to be the second most commonly (versus prostate in men and breast in women as most common) diagnosed cancer in Canada, excluding non-melanoma skin cancers, with an estimated incidence of 31,100 cases (about 13% of all new cancer cases) [3]. In Canada, approximately 1 in 15 men and women will develop lung cancer in their lifetime [4]. Further, in 2016 the overall incidence rate of lung cancer was estimated at 65.7 cases per 100,000; the incidence rates per 100,000 were estimated to be 12.8, 5.0, 12.0, and 30.5 for stages I, II, III, and IV, respectively. With its high incidence and poor prognosis (5-year overall survival rate of 22% during 2016–2017), lung cancer is the leading cause of cancer deaths in Canada. In Canada, 3-year survival rates for stages I, II, III, and IV are 71%, 49%, 22%, and 5%, respectively [1]. Internationally, 5-year survival rates have been reported for NSCLC in ranges (stage I 66–92%, II 47–60%, III 19–36%, IV 0–10%) because of different substages used in the IASLC Lung Cancer Staging Project [5].

Lung cancer is one of the most costly cancers [6], with an estimated cost to Canada’s healthcare system of $2 billion in 2020 [1]. Patient costs for lung cancer were estimated at approximately $22,000 per case (in 2014), including up to four lines of therapy for advanced disease, but not considering the indirect costs of cancer, including premature mortality and disability associated with this disease [7, 8].

### Risk factors and specific populations

The Canadian Population Attributable Risk of Cancer study found that 86% of lung cancer cases can be attributed to modifiable risk factors, with tobacco smoking contributing to 72% of cases [9]. Residential radon, asbestos, outdoor air pollution, physical inactivity, and certain occupational exposures such as construction, mining, and transportation [10] are other major factors linked to lung cancer. Nonmodifiable, independent risk factors include sex (20% higher in males in older ages [≥ 55 years] and in females at younger ages [45–54 years]), increasing age (e.g., incidence [per 100,000 for males and females, respectively] at 55–64 years are 118 and 108, at 65–74 years are 288 and 254 and at 75–84 years are 478 and 354), a personal or family history of lung cancer, a personal history of lung disease, and a weakened immune system [1]. Among those aged 45–84 years, the 5-year survival for lung cancer survival is 22% among females compared with 15% among males, regardless of histologic type, stage of disease, or province at diagnosis. Rates of survival decline with increasing age; 5-year survival was 35% for those aged 15–44 years and 21–22% for those aged 55–74 years [1]. Patients treated for other cancers with radiation therapy are also at an increased risk of developing primary lung cancer [11, 12].

From data from the Canadian Cancer Registry, across Canadian provinces lung cancer incidence (2012–2016), and parallel mortality (2013–2017), rates are highest in Nunavut, the Northwest Territories, Quebec (mortality data only), and to a lesser extent, in the Atlantic provinces [1]. This increased risk is likely due to a combination of risk factors (e.g., 63% and 35% of those ≥ 12 years smoke at least occasionally in Nunavut and the Northwest Territories, respectively [13]), diagnostic or treatment practices, and access to care [1]. Through systemic, economic, and geographic barriers, people living in rural or remote communities and/or having a lower income experience inequities in lung cancer incidence, being 77% more likely to smoke and receiving 6% more diagnoses at later stages, in access to care with 26% fewer curative surgeries, and in outcomes with 13–25% lower survival at 3 years, depending on stage [14]. Inequities in incidence and survival may be higher for lower income compared with geography [14]. A 2019 assessment of CT screening by the Institut National d’excellence en santé et services sociaux (INESSS) in Quebec, found that the socioeconomic and geographical gradients at risk could present organizational challenges to programs and create tensions between equality, equity of access, and service quality [15]. Although data on race and ethnicity is not collected by cancer registries, it is known that disparities in lung cancer incidence (e.g., 20–40% higher in First Nations people in Ontario [16]), mortality, and risk factors exist for Indigenous peoples in Canada with many contributing factors among a variety of social determinants of health [16–18]. First Nations, Inuit, and Métis populations have lower rates of cancer screening than non-Indigenous peoples in Canada [19].

### Risk prediction models

Various models exist to predict lung cancer incidence or prognosis. Using data from current and former smokers aged 55–74 years in two large screening trials (Prostate, Lung, Colorectal, and Ovarian [PLCO] Cancer Screening Trial and the National Lung Cancer Screening Trial [NLST]), the PLCO_M2012_ model is in widespread use and predicts cancer incidence over the following 6 years [20]. Risk factors in the model include higher age, black versus white race, lower socioeconomic status determined according to the level of education, lower body mass index, self-reported history of chronic obstructive pulmonary disease, personal history of cancer, family history of lung cancer, current smoking, smoking intensity (the average number of cigarettes smoked per day) and duration, and, in former smokers, shorter time since quitting. Using a lung cancer risk of 1.35% over 6 years, this model has higher sensitivity (83% vs. 71.1%) to detect lung cancer over 6 years than the eligibility criteria for the NLST trial (aged 55–74 years and ≥ 30 pack-year history and, if applicable, a quit time < 15 years) [20]. Although modeling studies indicate that the use of risk prediction models to select people for screening may increase screen-preventable deaths compared with a risk factors-based approach (e.g., NLST trial eligibility) [21], whether this translates into similar or greater net benefit from screening has not, to our knowledge, been investigated in a randomized clinical trial (RCT).

### Treatment approaches

The type, stage, and grade of lung cancer as well as one’s overall lung health will determine the type of treatment plan that is offered to individuals [22, 23]. For stages 0 and I NSCLC, surgical resection of the tumor is first-line therapy although radiation may be used instead or as adjuvant therapy if cancer is detected in the tumor margins. Chemotherapy may be provided after surgery for stage IB (having local invasion into visceral pleura) NSCLC. For stage II, extended pulmonary resection or chest wall resection may be recommended. Radiation alone or as an adjuvant may be an option, and other therapies (e.g., chemotherapy or possibly targeted anti-EGFR therapy or immunotherapy) often follow the initial therapy to lower the risk of recurrence. First-line treatment for stage III NSCLC is chemoradiation; surgery, possibly quite extensive and involving induction therapy with chemotherapy or chemoradiotherapy, may be an option for stage IIIa tumors, whereas additional therapies for stages IIIb and c are combinations of chemotherapy and/or therapies targeting tumor mutations (e.g., epidermal growth factor receptor inhibitors, anaplastic lymphoma kinase (ALK) therapy) or immunotherapies. Stage IV NSCLC is difficult to treat; treatment options are limited to chemotherapy, targeted therapies (e.g., monoclonal antibodies, tyrosine kinase inhibitors), and/or immunotherapies (e.g., immune checkpoint inhibitors). Radiation therapy may be offered if chemotherapy is not possible or to alleviate symptoms caused by the cancer. Surgery at stage IV NSCLC may be used to treat metastases to other organs but is not curative. Treatment advances have likely helped improve cancer survival over the past two decades. In Canada, age-standardized 5-year net survival increased by 4.5 percentage points (i.e., 14.5 to 19% survival) between 2002–2004 and 2012–2014 [24]; upwards of 10 percentage points in survival have been seen in Finland and Sweden between 2000–2004 and 2015–2019 [25]. In the USA, improvements in survival have been seen but are in part also attributable to improved access to care [26].

Given the rapid growth of SCLC and that diagnosis is usually in the later stages of the disease and often following metastases, treatment is usually systemic involving chemotherapy but may also include immunotherapy. Radiation may be used to prevent brain metastases or to shrink tumors to alleviate specific symptoms (e.g., trouble swallowing or breathing).

### Rationale for screening and screening approaches

A major reason lung cancers are diagnosed at a late stage is that early cancers are often asymptomatic [1]. Screening aims to detect earlier lung cancers when treatment is more likely to be curative. NSCLC is the main target for screening due to its higher prevalence and slower progression than SCLC. The most common screening modality used in current clinical practice is low-dose computed tomography (LDCT), largely based on positive results of the NLST trial showing superiority of three annual screens with LDCT over chest radiography in 53,454 smokers with at least a 30-pack year smoking history within the past 15 years; current or former) aged 55 to 74 years. This trial found 20% and 6% reductions in lung cancer and all-cause mortality, with numbers needed-to-screen 308 and 219, respectively [27]. This contrasts with evidence of lack of mortality benefit in the PLCO trial which compared four annual chest radiography screens versus usual care (no formalized screening program) for 154, 901 adults, 52% of which were current or former smokers, all aged 55 to 74 [28].

As with any screening, there are potential harms associated with lung cancer screening. There can be overdiagnosis, whereby screening identifies cancer that would have never caused harm, never progressed, progressed too slowly to cause symptoms or harm during a person’s remaining lifetime (e.g., a person dies from another cause), or that resolves spontaneously [29]. Major complications such as pneumothorax can occur from invasive diagnostic procedures such as bronchoscopy, surgical biopsy, thoracotomy, and mediastinoscopy; these adverse events may even occur in persons where the abnormality is found to be benign. There may also be psychosocial harms in anticipation of, or while undergoing, the screening procedure, and/or being told the screening result was indeterminate (e.g., requiring a repeat screen) or suspicious for cancer (e.g., requiring diagnostic testing) even when no cancer is present (i.e., a false positive). Additionally, imaging during screening may lead to incidental findings of possible pathology that can trigger a cascade of interventions that may lead to further complications or no improvement to one’s health (e.g., investigation of thyroid nodules).

Performance characteristics of the screening protocol affect false-positive (positive results on initial imaging among those later shown to not have lung cancer) and false-negative rates and therefore impact the ability of a program to detect cancers early while avoiding harms of investigations for benign lesions. The NLST trial used a simple classification system based on nodule size, and found a 23% rate of false positives, although positive tests included other incidental (e.g., cardiovascular) abnormalities. A more recent trial used a volume-based approach and found a false positive rate of 9.2%, comprised of indeterminate (requiring further screening) or suspicious lesions [30]. To standardize LDCT screening results reporting, the American College of Radiology developed and endorses the Lung-RADS™ classification system [31], which incorporates nodule characteristics (solid, part-solid, and non-solid) in addition to size. Lung-RADS v2022 category 0 “incomplete exam” requires recall. Lesions in Lung-RADS categories 1 and 2 are considered benign, whereas for category 3 (probably benign) and 4A (suspicious) lesions surveillance with LDCT in 6- or 3 months is suggested, respectively, and 4B (very suspicious) triggers diagnostic testing and/or tissue sampling. In Canada, a Nodule Risk Classification (NRC) system has been developed based on results from Pan-Canadian Early Detection of Lung Cancer (PanCan) nodule risk calculator, which incorporates nodule characteristics and location, in addition to age, sex, family history of lung cancer, and presence of emphysema to predict malignancy on a lung cancer screen [32, 33]. A review of the comparative accuracy between the PanCan and Lung-RADS found evidence of variable quality from six studies indicating that the PanCan model may perform better at determining which lung nodules identified by low-dose CT are cancerous compared to the Lung-RADS, though evidence from three other studies, also of variable quality, suggests that the risk calculators have similar diagnostic test accuracy [34]. RCTs examining clinically important outcomes using these or other alternative classification systems would be informative to determine if a lower degree of harm can be attained without compromising mortality benefits.

Also possibly impacting screening effectiveness, there are potential ways to refine patient selection criteria for screening such as using risk models incorporating demographic and clinical factors (e.g., PLCO_M2012_ 1.5% predicted risk of 6-yr lung cancer) [35] (as discussed above). Biomarkers such as autoantibodies, complement fragments, microRNAs, circulating tumor DNA, DNA methylation, or blood protein profiling are another option [36]. At least one RCT has compared the use of an autoantibody blood test to select people for CT screening versus usual care [37]. We are not aware of any trials that have been large enough to detect clinically important outcomes including mortality.

Currently, in Canada, there are no large-scale provincial organized screening programs for lung cancer although at least two provinces have either started (Ontario) [38] or plan to start (British Columbia) [39] organized programs of some scale. Some screening is occurring in several other provinces [40] and some provinces have pilot/demonstration projects underway [41]. Although primarily targeting radiologists, the Canadian Association of Radiologists supports shared decision-making involving information on the potential benefits and risks and on one’s personal risk for lung cancer, and recommends annual screening for people who have a 1.5% or higher risk (PLCO_M2012_ model) of developing lung cancer over the next 6 years (or at minimum meet the NLST smoking history criteria) until such time as they no longer meet eligibility criteria or develop health problems that substantially limit life expectancy or would preclude curative treatment [42].

### Patient values and preferences

Preferences for or against a screening strategy are influenced by the relative importance people place on the expected or experienced outcomes incurred [43–45]. Evidence on how people weigh the relevant outcomes is important to inform guideline panels when considering the balance of benefits and harms and determining whether this balance might vary across different individuals [46].

### Scope and purpose

In 2016, the Canadian Task Force on Preventive Health Care recommended screening adults 55–74 years of age who have at least a 30-pack-year smoking history and who smoked or quit smoking less than 15 years ago, annually for 3 years with low-dose CT (weak recommendation, low-quality evidence) [47]. They recommended not screening all other adults for lung cancer with low-dose CT, regardless of age, smoking history, or other risk factors (strong recommendation, very low-quality evidence), and that chest radiography with or without sputum cytology should not be used to screen for lung cancer (strong recommendation, low-quality evidence). Since that time, additional trials such as the NELSON [30], and longer follow-up [48] or additional data (e.g., harms [49]) from previously published trials of low-dose CT screening have been published that may improve understanding of the benefits and harms of screening and have the potential to change the direction or strength of the recommendations for screening. We will update the previous review conducted for the task force [50] on the benefits and harms of screening with CT with minor modifications; the task force is undertaking a separate reaffirmation process for the recommendation about screening with chest radiography. Although the benefits of screening will mostly rely on evidence from RCTs, we will also examine nonrandomized studies on the comparative effects between selection criteria used in RCTs and the use of risk prediction models for selection, as well as between trial-based and different nodule classification systems. Lastly, a systematic review of patients’ values and preferences will be conducted to allow fulsome insight for the task force when making judgments on the balance of benefits and harms. Findings from these reviews will be supplemented by input from patient and organizational stakeholders and by other sources of information on feasibility, acceptability, costs/resources, and equity to make recommendations for primary care providers [51]. The reviews will also serve as a comprehensive review for clinicians and other decision-makers on the effects of screening and relevant patient preferences.

## Methods

### Systematic review conduct

The Evidence Review and Synthesis Centre at the University of Alberta (JP, SR, LH) will conduct the systematic reviews on behalf of the task force following the research methods outlined in the task force methods manual [51]. We report this protocol and will conduct, and report, the reviews in accordance with current standards [52–56]. During protocol development, a working group was formed consisting of task force members (SK, DR, GT), with input from clinical experts (CF, NL, MM), and scientific support from the Global Health and Guidelines Division at the Public Health Agency of Canada (LAT, GTr, CG). The working group contributed to the development of the Key Questions (KQs) and PICOTS (population, intervention(s) or exposure(s), comparator(s), outcomes, timing [of outcome measurement], setting, and study design) elements.

Task force members made the final decisions with regard to the KQs and PICOTS. Task force members and clinical experts rated the proposed outcomes based on their importance for clinical decision-making, according to methods of Grading of Recommendations Assessment, Development, and Evaluation (GRADE) [57]. Ratings by the clinical experts were solicited to ensure acceptable alignment with the views of task force working group members, but task force members determined the final ratings. Final critical outcomes (rated at 7 or above on a 9-point scale) include lung cancer mortality, all-cause mortality, and overdiagnosis. The final important outcomes (rated 4–6) for inclusion are health-related quality of life (HRQoL), false positives, incidental findings, major complications and death from invasive procedures undertaken as a result of screening, and psychosocial harms from the screening process. The section on data extraction has details on how each of these outcomes will be defined. Further, since our calculations of overdiagnosis require estimates of cancer incidence we will extract and rate the certainty for data on incidence but it will not be an outcome considered during decision-making about overall recommendations for, or against, screening. We anticipate that there will be evidence on all-cause mortality, lung cancer mortality, and potential benefits of screening. We anticipate that there will be evidence of outcomes of harm including overdiagnosis of lung cancer, false positives, incidental findings, major complications and death from invasive procedures undertaken as a result of screening, and psychosocial harms. For incidental findings, we recognize that there may be some benefit to identifying some incidental findings; to help interpret these findings we will delineate as best possible between “clinically significant” and “any” incidental findings, and also perform analysis on certain specific incidental findings of interest. HRQoL may represent a benefit or harm of screening, depending on the direction of the effect. The final classification of benefit or harm for all outcomes will be based on the effects observed for different comparisons. Measures of values and preferences related to the critical and important outcomes were based on the GRADE methodology [43].

This version of the protocol was reviewed by the entire task force. Stakeholders (*n* = 16) reviewed a draft version of this protocol, and all comments were considered (Supplementary file 1). Throughout the conduct of the systematic reviews, we will document any changes to the protocol and we will report on these within the final reporting of the reviews.

### Key questions

1. What are the benefits and harms of screening for lung cancer in adults aged 18 years and older?
2. What is the relative importance people place on the potential benefits and harms of screening for lung cancer?
3.
  a What are the comparative benefits and harms of risk prediction models compared with trial-based criteria to identify eligibility for lung cancer screening?
  b What are the comparative benefits and harms of alternate nodule classification systems compared with nodule classification systems used in lung cancer screening trials?

### Eligibility criteria

Tables 1, 2, and 3 outline the eligibility criteria in terms of the PICOTS, for each KQ. For KQ1 we will include studies of adults in whom lung cancer is not suspected. Studies may enroll a general adult population or people meeting eligibility criteria associated with increased risk for lung cancer, as defined by authors. The population may include current, former, and second-hand smokers, as well as those with exposures to substances that may affect risk and other identified factors that may increase risk. Though CT, with or without any other lung cancer screening interventions (e.g., biomarkers), is the primary intervention of focus, we will include new RCTs published in 2015 or later of other screening modalities including chest radiography. Comparators can be either no screening or an alternative screening modality (e.g., chest radiography) or strategy (e.g., different eligibility criteria, classification of findings, screening interval). For benefits and overdiagnosis, we will only include RCTs because of the rigor of this design for overdiagnosis and the known availability of data with several years of follow-up [58]. For harms apart from overdiagnosis, where the effects are either rare and require large studies or only occurring/reported in the screening arm, we will include data from RCTs but also include nonrandomized and uncontrolled studies with some specific requirements as outlined in Table 1. We will proceed with examining the evidence on harms for a particular modality of screening when there is at least a low certainty of some benefit for one or more benefit outcomes from an RCT.

**Table 1:** Eligibility criteria for key question 1 on benefits and harms of screening for lung cancer with computed tomography

	Inclusion criteria	Exclusion criteria
Population	Adults ≥ 18 years old and not suspected to have lung cancer Specific populations of interest may include:	• Studies of people seeking care for symptoms of lung cancer or who are suspected of lung cancer • Studies of people who have been previously diagnosed with lung cancer • Studies of people younger than 18 years old • Study population includes > 25% individuals with recent abnormal screening result
	• Age	
	• Sex	
	• Smoking history (e.g., duration, pack-years)	
	• Race and ethnicity (e.g., First Nations, Inuit and Métis)	
	• Populations for which screening access and outcomes may be inequitable (e.g., LGBTQ + , low socioeconomic status, homeless)	
Intervention	Computed tomography (CT), with or without any other lung cancer screening interventions (e.g., biomarkers)	
	The focus is on screening interventions involving CT but we will include new RCTs (published in 2015 or later) of other screening modalities. Screening modalities (i.e., chest X-ray) examined in the last review (chest X-ray) will only be included if new RCTs are found	
Comparator	• No screening/placebo/minimally or non-active intervention (e.g., smoking cessation)	
	• An alternative screening strategy/protocol (e.g., use of risk-prediction model vs risk-factor based screening criteria, different screening interval or method to classify nodules)	
	• None (only for harm outcomes except overdiagnosis where a no screening arm is required)	
Outcomes (by importance as rated by the WG)	Benefits:	
	1. All-cause mortality*	
	2. Lung cancer mortality*	
	3. Health-related quality of life	
	If there is trial evidence showing at least low certainty of benefit for 1 + of the above outcomes, then:	
	Harms:	
	4. Overdiagnosis of lung cancer*	
	5. Major complications or morbidity from invasive testing as a result of screening	
	6. Death from invasive testing as a result of screening	
	7. False positives	
	8. Psychosocial consequences of the screening process (i.e., before or right after screening, receiving a false positive result)	
	9. Incidental findings	
	*Rated as critical outcomes; others rated as important	
Timing	No limitation on the duration of follow-up, except for FPs where ≥ 12 months follow-up after the screening result is required in ≥ 80% of participants (if there is no evidence found meeting this criteria we will accept ≥ 6 months follow-up)	
	Longest follow-up will be used for mortality, quality of life, and overdiagnosis unless there has been substantial (> 20%) contamination by the control group receiving screening	
Delivery setting	Any setting relevant to primary care	
	Countries rated as very high on the Human Development Index 2019 [59]	
Study design & publication type	• RCTs (parallel designs using individual or cluster randomization)	• Studies using modeling or simulation, or retrospectively applying differing screening criteria • Editorials, commentaries • Case reports and series (i.e. all participants have the outcome)
	• With the exception of overdiagnosis, for harms nonrandomized studies are also eligible. For false positives, the study must use similar selection criteria and methods of determining a positive result (e.g., diameter, volumetric) as used in an RCT. If a method used in an RCT is compared with another method in a nonrandomized study these will be considered for in KQ3. For psychosocial harms, there must be at least a within-group comparison relevant to the outcome e.g., before and after the screening test, between people with a negative vs. a positive test	
	Note: nonrandomized studies comparing the benefits of screening selection criteria or the nodule classification systems used in the RCTs with the use of a risk prediction model or a different nodule classification system will be considered in KQ3	
	Journal articles Letters, abstracts, and grey literature (e.g., government reports, results in trial registries) if information on study design (e.g., eligibility criteria, participant characteristics, presentation of scenarios) is sufficient to assess study and results are confirmed as final (accessible online or via author contact)	
Language of full text	English or French	
Dates of publication	Any date

**Table 2:** Eligibility criteria for key question 2 on the relative importance people place on the potential benefits and harms from screening for lung cancer

	Inclusion	Exclusion
Population	Adults aged 18 years and older	Expert or healthcare providers (doctor, nurse) acting as proxies for patients and the public
	Specific populations of interest may include the following:	
	• Age	
	• Sex	
	• Smoking history	
	• Race or ethnicity (e.g., First Nations, Inuit, and Métis)	
	• Populations for which screening access and outcomes may be inequitable (e.g., LGBTQ + , low socioeconomic status, homeless)	
	• For health-state utility studies: cancer detection via screening vs. clinical presentation; stage/severity of cancer (i.e., curative vs. palliative/non-metastatic vs. metastatic)	
Exposures	a) Direct measurements:	
	Utility-based measurements (e.g., health state utilities, trade-offs between outcomes)	
	i. Experience with outcome/health state (as per KQ1, adding different stages/severities of cancer)	
	ii. Exposure to clinical scenarios about the outcome(s)	
	iii. Exposure to choice sets or other risk exercises (e.g., trade-offs, balance sheet, ranking) with differing risks/magnitudes of effects on benefits versus harms from screening *(must contain 1* + *benefit and 1* + *harm)*	
	Quantitative non-utility studies (e.g., simple ratings, rankings, or trade-offs between 1 + benefit and 1 + harm)	
	i. Any exposure	
	b) Indirect measurements (allowing inferences about how many people perceive benefits as more important than harms & acceptability of screening; not only in the context of critical outcomes):	
	i. Exposure to estimates of effect from screening for 1 + benefit and 1 + harm (e.g., decision aids)	
Comparisons	a) Utility-based measurements:	Studies measuring utilities during cancer treatments that are not considered a standard first-line of care for a representative sample, by stage of cancer (will gather clinical input as needed)
	i. Healthy/usual state without outcome (may include screen-negative patients)	
	ii. Different outcome/health state (e.g., false positives vs. overdiagnosis; includes studies comparing different stages/severity of cancers)	
	iii. No comparison, if no other studies for the outcome comparison	
	b) Quantitative non-utility studies and indirect studies	
	i. No comparison	
	ii. Comparison with another screening strategy (e.g., having different magnitude of effects; based on comparisons evaluated in evidence found for KQ1)	
Outcomes	a) Direct measurements:	
	i. Health-state utility values; health states may include different stages/severities of lung cancer and nodules to help estimate the utility of an overdiagnosed case	
	ii. Other utility scores (e.g., trade-offs, magnitude of coefficients/utility weights in models for each outcome)	
	iii. Non-utility, quantitative information about the relative importance of different benefits and harms (ratings, rankings)	
	b) Indirect measurements:	
	i. Inferences about how many people perceive benefits as greater than harms	
	ii. Preference for or against screening (screening attendance, intentions, or acceptance) or preferred screening strategy based on different outcome risk descriptions (e.g. using decision aids)	
	c) Measures of variability for all of the above (e.g., 95% CIs, proportion unwilling to trade any benefit for harm, proportion undecided from decision aids vs. those confident in decision for vs. against)	
Timing	Measured immediately after experiencing outcome/diagnosis (to 3 months) and longer-term (e.g., after investigations and treatment)	
Setting	Any setting in Very High Human Development Index countries [59]	
Study design and publication status	Any quantitative study design	Editorials
	Journal articles Letters, abstracts, and grey literature (e.g., government reports, results in trial registries) if information on study design (e.g., eligibility criteria, participant characteristics, presentation of scenarios) is sufficient to assess study and results are confirmed as final (accessible online or via author contact)	
Language	English or French	
Publication date	2012–present

**Table 3:** Eligibility criteria for key question 3 on the comparative effects between (a) trial-based selection criteria and use of risk prediction models, and (b) trial-based nodule classification and different nodule classification systems

	**Inclusion**	**Exclusion**
Population	Adults aged 18 years and older are not suspected to have lung cancer	Studies that focused on people under age 18 or that targeted adults 18 years and older who were either suspected of having lung cancer or were previously diagnosed with lung cancer
Intervention(s)	For risk prediction models used for screening eligibility:	Risk prediction models that include factors (laboratory tests, other assessments) that are not available or feasible in primary care Risk prediction models are used for screening eligibility that incorporate nodule characteristics
	Externally validated risk prediction models intended for identifying persons who may benefit from screening	
	The subsequent screening process must be similar to trials included in KQ1 (e.g. LDCT, nodule classification)	
	Note: The purpose of the study may be to provide external validation	
	For nodule classification systems: any nodule classification system. Other elements of screening must be similar to the trial included in KQ1	
Comparison(s)	For risk prediction models:	
	Trial-based eligibility criteria (similar to that used by trials included in KQ1 (e.g., NLST, NELSON))	
	For nodule classification systems:	
	Nodule classification system is similar to those used by trials included in KQ1 (e.g., NLST, NELSON)	
Outcomes	Benefits:	False positive selections (individual is positive for “increased risk” using model [i.e. is selected for screening with CT or other modality] but does not get cancer or die from lung cancer during follow-up)
	1. All-cause mortality*	
	2. Lung cancer mortality* 3. Health-related quality of life Harms: 4. Overdiagnosis of lung cancer* 5. Major complications or morbidity from invasive testing as a result of screening 6. Death from invasive testing as a result of screening 7. False positives 8. Psychosocial consequences of the screening process (i.e., before or right after screening, receiving a false positive result) 9. Incidental findings *Rated as critical outcomes; others rated as important See Table 1. Note: outcome may be reported as a relative measure between benefit(s) and harm, e.g., number of overdiagnosed cancers per prevented death	
Delivery setting	Any setting relevant to primary care	
Study design(s) and publication status	Nonrandomized clinical trials, prospective or retrospective controlled observational studies (including analyses using data from screening arms in RCTs), modeling studies	• RCTs (captured in KQ1) • Editorials • Commentaries • Case reports and series
	Journal articles	
	Letters, abstracts, and grey literature (e.g., government reports, results in trial registries) if information on study design (e.g., eligibility criteria, participant characteristics, presentation of scenarios) is sufficient to assess study and results are confirmed as final (accessible online or via author contact)	
Countries	Studies conducted on populations from countries categorized as “Very High” on the 2016 Human Development Index (as defined by the United Nations Development Programme)	Studies conducted on populations from countries not categorized as “Very High” on the 2016 Human Development Index (as defined by the United Nations Development Programme)
	Priority will be given to studies relevant to the Canadian context	
Language of full text	English or French	Languages other than English and French
Dates of publication	2012 (publication of first RCT showing the benefit of lung cancer screening was Aug 2011)	

For KQ2 on values and preferences, individuals may or may not have experienced lung cancer or one or more of the critical or important outcomes of interest. Study designs may be any quantitative design measuring preferences for outcomes either directly such as health-state utilities or trade-offs, or indirectly, hence allowing inferences about relative values based on the degree of acceptance of screening given scenarios with estimates of the expected benefits and harms.

For KQ3, we will include nonrandomized studies comparing the benefits and harms between randomized trial (included in KQ1) (KQ3a) selection criteria or (KQ3b) nodule classification systems and (KQ3a) selection based on (externally validated) risk prediction models or (KQ3b) different nodule classification systems. The selection criteria should be similar to, but does not need to be identical to that used in a trial included in KQ1; for instance, if the minimum age differs by 2–3 years, the review team will use clinical input to decide eligibility. RCTs of these comparisons will be included in KQ1.

For the most relevance to Canada, we will only include studies conducted in countries listed as very high according to the Human Development Index [59] and having full texts in English or French. For KQs 2 and 3, we will limit studies to those published during or after 2012. Based on clinical input and working group discussion, utility-based outcomes will have changed over time because treatments and their impact have changed quite dramatically, and the best indirect measurements, for example, based on decision aids, would be based on contemporary estimates of the effect of CT screening since the publication of NLST trial in 2011. This date also represents the publication of the major risk prediction models and the emergence of studies comparing different screening protocols.

### Searching the literature

For KQ1 we will locate all full texts from the previous task force review [50]. The previous review’s final search date for studies was March 31, 2015 [50], so for this KQ, we will search Medline and Embase, via Ovid, and Cochrane Central including the Central Register for Controlled Trials from 2015 onwards. The previous searches for benefits and harms were modified slightly to increase their sensitivity, with a more sensitive filter applied for RCTs, and broaden their scope, such as adding controlled vocabulary and keywords for incidental findings and psychosocial harms into the harms search. For KQ2 we will search Ovid MEDLINE (1946–), Scopus, and EconLit from 2012 using two searches: one for utility-based studies, focusing on relevant preference-based instrument/methodology terms and the relevant outcomes as well as lung cancers and nodules to help estimate the utility of an overdiagnosed case) and another for decision-making/acceptance/attitudes about lung cancer screening. For KQ3, we will search Medline and Embase; for MEDLINE we will rely on the 2019 search performed by the authors of a review on this question to inform the United States Preventive Services Task Force (and screen all of their studies for eligibility) [60] and update the search to present using a de novo search strategy, and for Embase we will run the de novo search from 2012. Searches were developed in collaboration with an information specialist and peer-reviewed by another using the PRESSS 2015 checklist [61]. The final Medline searches are located in Supplementary file 2. We will scan reference lists of included studies and relevant reviews. We will search ClinicalTrials.gov and the World Health Organization International Clinical Trials Registry Platform for results data for published and unpublished trials (past 2 years) of lung cancer screening. Where studies are only reported in conference abstracts or trial registries, the first authors will be contacted by email with two reminders over 1 month to confirm results are final and see if full study reports are available. Any unpublished data will be subject to sensitivity analysis if included.

We will export the results of database searches to an EndNote library (Clarivate Analytics, Philadelphia, USA, 2018) for record-keeping and will remove duplicates. We will document our supplementary search process, for any study not originating from the database searches, and enter these citations into EndNote individually. We will update electronic database searches for all KQs within 12 months of the task force guideline publication.

### Selecting studies

Records retrieved from the database searches will be uploaded to DistillerSR (Evidence Partners Inc., Ottawa, Canada) for screening. For all citations retrieved from the database searches, two reviewers will independently screen all titles and abstracts using broad inclusion criteria. Full texts of any citation from the search considered potentially relevant by either reviewer will be retrieved. Two reviewers will independently review all full texts including the studies from the previous reviews against a structured eligibility form, and a consensus process will be used for any full text not included by both reviewers. If necessary, a third reviewer with methods or clinical expertise and/or author contact will be used to arbitrate decisions. The screening and full-text forms will be pilot-tested with a sample of at least 100 abstracts and 20 full texts, respectively. Screening studies located from reference lists, trial registries, and websites will be conducted by one experienced reviewer, with two reviewers reviewing full texts. We will document the flow of records through the selection process, with reasons provided for all full-text exclusions, and present these in a PRISMA flow diagram [55] and appended excluded studies list. When data from multiple reports from the same trial are used in the review for results of mortality, HRQoL, or cancer incidence (for estimating overdiagnosis), we will consider the report from which we collected lung-cancer mortality data to be the primary (included) publication for citation but will cite the others as companion papers. When we are using data on harms only in the screening arm within a trial, we will consider the study a different, uncontrolled study, and cite the report we used for the data unless it is the primary publication.

### Data extraction

We will rely on data extraction from the previous review team, as able and suitable. For this data and for all data from new studies, one reviewer will extract data and another will verify all data for accuracy and completeness. Results data for the critical outcomes in KQ1 will be extracted in duplicate, with decisions based on consensus or arbitration by a third reviewer. Each data extraction form will be piloted with a sample of at least five studies.

Sufficient data will be collected to allow examination of the homogeneity and similarity assumptions for meta-analysis, for description and possible analyses on specific populations (see Tables 1 and 2), and for assessment of the risk of bias. The main data items include the study characteristics (i.e., year and country of conduct, eligibility criteria, sample size eligible and enrolled, setting of recruitment, trial/study design, methods for randomization, concealment and blinding); population (i.e., age, sex, details on any population(s) requiring equity considerations [e.g., LGBTQ + , low socioeconomic status], race or ethnicity, personal history of lung disease, family history of lung cancer, smoking history [past, current, pack-years, others], health state including diagnosis, stage of cancer and treatment received [for KQ2 preference studies]); intervention and comparator (e.g., interval, rounds, dose, classification of nodules, description of usual care and any adjuvant therapies including smoking cessation advice etc.) (for effectiveness) or exposure (e.g., instrument, measurement of tariffs, scenarios used, estimates of effects of screening, any specified durations of health states) (for KQ2 preference studies); outcomes (definitions, ascertainment, methods to determine cause of death, timing of data collection, tool with range of values for patient-reported outcome measures); number screened at each round or during usual care; cumulative number of cancers diagnosed (including the proportion diagnosed based on screening results); details of an adjusted analyses in nonRCTs, results (numerator and denominator for each outcome; see details below); funding source; data supporting missing outcomes or analyses.

For most of the outcomes in KQ1, the denominators will be the population enrolled in the relevant arm/group(s) in the study (i.e., intention-to-treat). One exception is psychosocial harms where sub-populations (e.g., those receiving a positive screening result) will be considered. Another exception is for overdiagnosis. We will calculate estimates of overdiagnosis by the relative [58] and absolute risk of cumulative lung cancer incidence through follow-up in the screening compared with no screening group, and by the excess incidence of cancer from screening among those (i) having cancer diagnosed in the screening arm, and (ii) having cancer diagnosed through screening in the screening arm.

For mortality outcomes and overdiagnosis (using cancer incidence), we will use crude data on the cumulative number of events from the longest follow-up time point unless there has been substantial contamination after a previous time point (> 20% no screening group receiving screening). For incidence rates for use when calculating overdiagnosis there must be follow-up beyond the active phase of the screening. For HRQoL, we will extract the mean baseline and endpoint or change scores (at longest follow-up without substantial contamination), standard deviations (SDs), or other measures of variability, and the number analyzed in each group. For the outcomes of major complications or morbidity (requiring hospitalization or medical intervention), and mortality, from invasive testing as a result of screening, we will use counts of the number of people having one or more events (not the total number of events) among those who later receive a negative diagnosis (false positives) and among anyone receiving the invasive testing (those with cancer and false positives). For incidental findings, we will extract all data on the number of people with an incidental finding (“any” as well as “actionable/clinically significant” [including definitions]), unless only number of incidental findings is reported, and details of the incidental findings, that is, the organ system involved and whether it resulted in referral for additional testing. For false positives and psychosocial harms from screening, we will examine results for anyone receiving a recommendation for early recall (e.g., indeterminate result with repeat CT screening at 3 or 6 months) or for a diagnostic follow-up (e.g., result suspicious of lung cancer) as well as only for those recommended to have diagnostic follow-up. Other definitions of false positives will be considered. We will record the proportion of people receiving at least one false positive result over all screening rounds and the average number of false positives during the active screening phase of the study. Because false positives can take many months to resolve, we will make sure to report who is included in the rates, such as whether there are many people missing from the result because of unresolved diagnostic information. Results considered consistent with the outcome of psychosocial harms include data from patient-reported outcome measurement tools/questionnaires on symptoms of anxiety, depression, distress, and concern about lung cancer; if composite scores meeting these concepts are available we will use these and not subscales. Single-question items are not eligible. Subscales of overall HRQoL scales (e.g., mental health) will be considered to measure psychosocial harm if other tools measuring the same symptoms are not reported. For this outcome we will extract data at all time points where there is measurement, during the active phase of screening, to primarily capture these harms from undertaking screening itself and from receiving a false positive result.

When only relative effects/ratios between groups are reported instead of raw counts and intention-to-treat is not used, we will rely on results from last-observed carry-forward or, if necessary, per protocol/completer approaches, as reported. For missing results data for any outcome, including measures of variance, we will contact authors by email with two reminders over 1 month. If not received, as possible we will compute missing SDs or standard errors (SEs) from other study data, or as a last resort, impute based on other studies in the review. When computing SDs for change from baseline values, we will assume a correlation of 0.5 [62], unless other information is present in the study that allows us to compute it more precisely. We will use available software (i.e., Plot Digitizer, http://plotdigitizer.sourceforge.net/) to estimate effects from figures if no numerical values are provided. If cross-over trials are included, we will limit the data extraction to the first period of the study, prior to the cross-over.

For KQ2 health-state utilities, data using the most commonly used measurement tool (e.g., EuroQol 5 Dimensions [[EQ-5D]), using tariffs from the same country, at the earliest time point after baseline (or diagnosis) will be prioritized for analysis [63] though we will extract all data meeting our criteria in Table 2.

For data from non-randomized studies, we will rely when possible on results adjusted for potential confounders.

### Risk of bias

For RCTs we will use the Cochrane risk of bias (ROB 2.0) tool, assessing the effect of assignment to the intervention for each relevant outcome (cancer-specific and all-cause mortality, cancer incidence [to calculate overdiagnosis] and HRQoL) [64]. For nonrandomized studies (including single-arm data on harms from RCTs), we will use the checklists, as applicable, from Joanna Brigg’s Institute [65], with the exception of preference-based studies where will use items as per GRADE guidance, about the choice/selection of representative participants; appropriate administration and choice of instrument; analysis and presentation of methods and results; instrument-described health state presentation, of all relevant outcomes and valid with respect to health state; patient understanding; and subgroup analysis to explore heterogeneity [43]. Major potential confounders of interest are age, sex, and smoking history.

Two reviewers will independently assess the studies and come to a consensus on the final risk of bias assessment for each question using a third reviewer where necessary. Each risk of bias tool will be piloted with a sample of at least five studies, using multiple rounds until agreement on all elements is high. These assessments will be incorporated into our assessment of the risk of bias across studies when rating the certainty of the evidence for each outcome using GRADE.

### Data analysis

When two or more outcome comparisons are sufficiently similar, we will pool their data. The decision to pool studies will not be based solely on statistical heterogeneity; the *I*^2^ statistic will be reported, but it is recognized that the *I*^2^ is influenced by the number of studies and the magnitude and direction of effects [66]. Rather, we will rely on interpretations of the clinical (related to our PICOTS, e.g., the definition of positive screening result) and methodological differences between studies. For pairwise meta-analysis, when there are large differences in trial sizes and potential publication bias or within-study bias in smaller studies [67], our main analyses will employ a fixed-effects model using Stata. If these factors are not apparent we will use a random effects model. We will use the DerSimonian Laird method unless events are rare (< 1%) where we will pool odds ratios (ORs) using Peto’s method or (if zero events) the reciprocal of the opposite treatment arm size correction. For dichotomous outcomes, we will analyze and report data using risk ratios (RRs) and their 95% confidence intervals (95% CIs) unless ORs are used with the Peto method, where we will convert ORs to RRs using control event rates. For continuous outcomes, we will report a pooled mean difference using changes scores, when one measurement tool is used. We will use a standardized mean difference when combining two or more outcome scales measuring similar constructs based on clinical input. If suitable, we will transform the results to either a mean difference or ratio to assist interpretation [68]. For pooling proportions which we anticipate for most harms, we will apply suitable transformation (logit or arcsine) depending on the proportions of events and use a random effects model [69]. Pooling of mean health state utilities will use a random-effects model with weighting by the inverse of variance. If we are not able to use a study’s data in a meta-analysis (e.g., only *p* values are reported), we will comment on these findings and compare them with the results of the meta-analysis. Analyses will be performed using Microsoft Excel, Review Manager (version 5.3), and STATA (version 14.2 or higher). Relative effects will be transformed into absolute effects using the pooled control events rates across the included studies. Based on clinical input, we may also assume one or more different control/baseline rates for estimating absolute effects in a lower and/or higher-risk population. For mortality outcomes having statistically significant effects, we will calculate the number needed to screen and its 95% CI.

If meta-analysis is not undertaken, we will synthesize the data descriptively. We will use various techniques as described for narrative syntheses, such as creating an overall synopsis of each study, including their characteristics and reported findings, and describing relationships within and between studies focusing on our exposure subgroups and outcome comparisons of interest and other factors such as methodological quality [70].

#### Unit of analysis issues

In the event of the inclusion of cluster-randomized trials, we will take appropriate measures to avoid unit-of-analysis errors when reporting their findings and/or incorporating them into meta-analysis [71]. When available, we will use the intracluster correlation coefficient reported in the trial to apply a design effect to the sample size and number of events in each of the treatment and control groups [72]. If not reported, we will use an external estimate from similar studies. We will clearly identify cluster-randomized trial data when it is included in meta-analysis with individually randomized trials.

#### Assessment of heterogeneity

When statistical heterogeneity in the direction of effects is seen across studies, we will conduct subgroup (stratified) analyses, using variables associated with the population (specific populations in Tables 1, 2, and 3), the intervention (e.g., screening interval), or exposure (e.g., scale measuring utilities), or the follow-up duration, and/or sensitivity analysis removing the high risk of bias studies, data from unpublished studies, or studies for which we needed to impute measures of variance or adjust for clustering. Subgroups will be tested for statistical significance and the credibility of the results interpreted using available guidance [73]. We will also extract results from within-study analyses related to our specified variables of interest.

#### Small study bias

When meta-analyses of trials contain at least 10 studies of varying size, we will test for small study bias visually by inspecting funnel plots for asymmetry and statistics via Egger’s test (continuous outcomes) [74] or Harbord’s test (dichotomous outcomes) [75].

### Rating the certainty

We will use GRADE methods to assess the certainty of evidence for all outcomes [43, 45, 46, 76]. In cases where studies of interventions cannot be pooled in a meta-analysis, we will use guidance for rating the certainty of evidence in the absence of a single estimate of effect [77]. Two reviewers will independently assess the certainty of evidence for each outcome and agree on the final assessments. A third reviewer will arbitrate if necessary.

We will assess the certainty of evidence (very low, low, moderate, or high) based on five domains: study limitations (risk of bias), inconsistency of results, indirectness of evidence, imprecision, and reporting biases (small study bias or missing outcome data). For cancer incidence, mortality, and HRQoL, RCTs will start at high certainty and nonrandomized studies will start at low certainty. False positives, incidental findings, and complications and mortality from invasive procedures resulting from screening are most often only reported for the screening group (even in RCTs), and therefore certainty will start at low (with the possibility to rate up for assumption of large effects) for the studies reporting these outcomes except when a comparison between different screening approaches in a randomized design is the focus. For psychosocial outcomes, the initial certainty will depend on the study design with RCTs having data for these outcomes in both arms starting at high and other studies starting at low certainty. Rating will be considered when relying on nonrandomized studies if there are no serious concerns about the other domains [78]. Studies measuring preferences and health state utilities will start at high certainty. Unless the outcome is measured using an instrument (e.g., for HRQoL) that has a known minimally important difference around which to base our conclusions and certainty, we will initially apply a minimally contextualized approach whereby we will rate our certainty in the direction of effect (i.e., relative to the null effect) rather than a particular magnitude of effect [76]. Rather than statistical significance, a threshold of a minimal effect (i.e., to determine if results very close to the null are of little to difference) may be chosen before the task force reviews the results. Upon examining the findings, the task force may decide to adopt a partially or fully contextualized approach using one or more thresholds (e.g., for small and moderate magnitudes of effect) and consider multiple outcomes simultaneously. In such cases, the assessment of heterogeneity (i.e., by magnitude) and certainty ratings will be revised accordingly.

We will prepare GRADE Summary of Findings tables, by outcome for each comparison, including explanations for all decisions.

### Task force involvement

The task force and clinical experts will not be involved in the selection of studies, extraction of data, appraisal of the risk of bias, or synthesis of data, but will contribute to the interpretation of the findings and comment on the draft report. Clinical experts and/or task force members may be called upon to contribute to the identification of thresholds and the certainty of evidence appraisals, e.g., to interpret the directness (applicability) of included studies to the population of interest for the recommendation.

## Discussion

The review will be published in an open-access journal and reported using a standard checklist for systematic reviews [55]. The results section of the review will include a description of the flow of literature and characteristics of all studies, results of all analyses, including planned subgroup and sensitivity analyses as well as the summary of finding tables incorporating assessment of our certainty in the evidence. In the discussion, we will summarize the main findings and their implications, compare our findings to other systematic reviews, and discuss the limitations of the review and the available literature. The results will be used by the task force for developing recommendations about screening for lung cancer with low-dose CT. It will also serve as a comprehensive review for clinicians and other decision-makers on the effects of screening and relevant patient preferences.

### Protocol amendments

We will report on any deviations from the protocol within the final manuscript.

### Supplementary Information

**Additional file 1.** Responses to stakeholder reviews of the draft protocol.

**Additional file 2.** MEDLINE Search Strategies.

## Data Availability

Not applicable.

## Abbreviations

CI: Confidence interval
CT: Computed tomography
EQ-5D: EuroQol-5 dimension
GRADE: Grading of Recommendations Assessment, Development and Evaluation
HRQoL: Health-related quality of life
KQ: Key question
NLST: National Lung Cancer Screening Trial
NRC: Nodule Risk Classification
NSCLC: Non–small cell lung cancer
OR: Odds ratio
PHAC: Public Health Agency of Canada
PICOTS: Population, interventions, comparators, outcomes, timing, setting
PLCO: Prostate, Lung, Colorectal, and Ovarian Cancer Screening Trial
RCT: Randomized clinical trial
RR: Risk ratio
SD: Standard deviation
SCLC: Small cell lung cancer
UC: Usual care
