# Supplementary material for: Screening for lung cancer with computed tomography: protocol for systematic reviews for the Canadian Task Force on Preventive Health Care
Source: Syst Rev. 2024 Mar 16;13:88. doi: 10.1186/s13643-024-02506-3 (PMC10943889; doi:10.1186/s13643-024-02506-3)
Supplement: Supplementary file 1 — Additional file 1. Responses to stakeholder reviews of the draft protocol. [file 13643_2024_2506_MOESM1_ESM.docx]

**Screening for Lung Cancer with Computed Tomography: Systematic Reviews for the Canadian Task Force on Preventive Health Care**

**Supplementary file 1**

**Responses to stakeholder reviews solicited by Canadian Task Force on Preventive Health Care**

| **Reviewer and (at first instance) affiliation** | **Yes/No** | **Comments** | **Response** |
| --- | --- | --- | --- |
| **Question 1: The Task Force is committed to creating guidelines that enhance equity.**  **Are there additional specific populations that should be considered for this topic, and do you have any concerns or suggestions regarding how specific populations of interest within the background and PICO table have been described?** | | | |
| Houda Bahig, Canadian Association of Radiation Oncology | No | Very complete. | Thank you. No changes requested |
| Jim Boulanger, Institut national d’excellence en sante et en services sociaux (INESSS) | No | The population considered seems adequate for the evaluation questions. | Thank you. No changes requested |
| Carole Dennie, Canadian Association of Radiologists | No |  | Thank you. No changes requested |
| Craig Earle, Canadian Partnership Against Cancer | No | Appropriately calls out Indigenous peoples as the main subpopulation of interest in Canada | Thank you. No changes requested |
| Elizabeth Holmes, Canadian Cancer Society | Yes | Ensures Black individuals and other racialized communities are included. Include sexual orientation (due to smoking rates of LGBTQ+ communities) | Thank you. To better account for sexual orientation as well as other specific populations requiring equity considerations we have revised our specific populations of interest (see Tables 1 and 2) to add “Populations for which screening access and outcomes may be inequitable (e.g., LGBTQ+, low socioeconomic status, homeless)”, though smoking history will also be considered across all populations. Equity is an important element that may not be captured in the reviews but is part of the additional considerations the Task Force considers using other evidence/information such as stakeholder comments. |
| Olivia Kulbak, Canadian Cancer Society | Yes | Ensures Black individuals and other racialized communities are included. Include sexual orientation (due to smoking rates of LGBTQ+ communities) | Thank you. To better account for sexual orientation as well as other specific populations requiring equity considerations we have revised our specific populations of interest (see Tables 1 and 2) to add “Populations for which screening access and outcomes may be inequitable (e.g., LGBTQ+, low socioeconomic status, homeless)”, though smoking history will also be considered across all populations. Equity is an important element that may not be captured in the reviews but is part of the additional considerations the Task Force considers using other evidence/information such as stakeholder comments. |
| Stephen Lam, BC Cancer Foundation | Both yes and no are checked | To determine screening eligibility, we need to address disparity related to race, sex and socioeconomic status in addition to First Nations, Metis and Inuit | Thank you. To better account for specific populations requiring equity considerations we have revised our specific populations of interest (see Tables 1 and 2) to add “Populations for which screening access and outcomes may be inequitable (e.g., LGBTQ+, low socioeconomic status, homeless)”. Equity is an important element that may not be captured in the reviews but is part of the additional considerations the Task Force considers using other evidence/information such as stakeholder comments. |
| Stan Marchuk, Nurse Practitioners’ Association of Canada | Yes | No comment | Thank you. No changes requested |
| Wade Norquay, Department of Health and Wellness, PEI | Yes | If at all possible include those in the LGBTQ2+ communities | Thank you. To better account for specific populations requiring equity considerations we have revised our specific populations of interest (see Tables 1 and 2) to add “Populations for which screening access and outcomes may be inequitable (e.g., LGBTQ+, low socioeconomic status, homeless)”. Equity is an important element that may not be captured in the reviews but is part of the additional considerations the Task Force considers using other evidence/information such as stakeholder comments. |
| Larry Pan, PEI Cancer Treatment Centre | Yes | Equity, diversity and inclusion goals may be challenging to achieve through the current methodology. The Indigenous population (First Nations, Inuit, and Metis) may not be well represented in traditional health research studies. | Thank you. We recognize this potential limitation of the evidence reviewed for these reviews. Equity is an important element that may not be captured in the reviews but is part of the additional considerations the Task Force considers using other evidence/information such as stakeholder comments. |
| Diana Sanchez-Ramirez, Manitoba Lung Association | No | PICO Tables 1 and 2 stated race and ethnicity as one of the inclusion criteria, indicating that the authors intended to include multiple populations. From what is described in the document and used as an example in the PICO questions, the interest in involving studies related to the group of indigenous people is clear. However, I would also like to highlight the importance of including other racial and ethnic groups considering the great diversity of the Canadian population enriched by the constant migratory flow.] | Thank you. We will consider a wider range of racial and ethnic groups. |
| Ambreen Sayani, Canadian Partnership Against Cancer | Neither checked off | Inequities in lung cancer risk, outcomes and mortality patterns are described in the protocol. However, how this will relate to any of the key questions or analysis is unclear. For example, a key question in my mind is, what are the population-level harms of introducing lung cancer screening when both risk and uptake are inequitable in the described populations? I realise that may be beyond the scope of the review – however, there are serious risks of making lung cancer an even more inequitable disease and potentially widening the socioeconomic gradient in lung cancer mortality between population groups based on unequal participation in screening. | Thank you for the comments. Equity is an important element that may not be captured in the reviews but is part of the additional considerations the Task Force considers using other evidence/information such as stakeholder comments. |
| Howard Tracer, United States Preventive Services Task Force | No | No, but please see my comments under Q2 re. Black persons and risk of lung cancer, and possible lack of data (or risk prediction models) relevant to the disparities in lung cancer incidence and mortality you describe in Indigenous populations in Canada. | Thank you. |
| **Question 2: Do you have any comments or suggestions related to the eligibility criteria (interventions, comparisons, outcomes) for the research questions? (see relevant tables *[Tables 1-3]*)** | | | |
| Bahig | No |  | Thank you. No changes requested |
| Boulanger | No | Eligibility criteria are clearly described. | Thank you. No changes requested |
| Dennie | No |  | Thank you. No changes requested |
| Earle | No | Looks good | Thank you. No changes requested |
| Holmes | No | N/A | Thank you. No changes requested |
| Kulbak | No | N/A | Thank you. No changes requested |
| Lam | Yes | General Comment: LDCT screening is for people above 50 or 55 years of age. Why do we start at ≥ 18 years?  **Table 1.**  1. Many biomarkers have been proposed, but none have survived robust external validation or have been translated into routine clinical use (including reference 35, Sullivan et al). Should this be excluded in the review?  2. Comparator: Lung cancer screening is a risk-based screening. One can compare a risk factor-based approach (age and pack years) versus a risk model-based approach (PLCOm2012) to determine screening eligibility. If we consider risk model as “pre-screening”, risk factor approach should be considered the same.  3. It is important to assess the benefits and harms of screening. However, the focus of Table 1 and Table 3 needs to be separate. Benefits and harms based on screening eligibility criteria are different from benefits and harms related to diagnostic workup and treatment. If we select participants with sufficient lung cancer risk, the potential benefits will outweigh the potential harms. The harms listed in Table 1 only occur after a screening LDCT has been done and the next step recommendation leads to a diagnostic work-up or surgery. Screening low risk individuals is how we generate overdiagnosis, risk of radiation exposure and complications from invasive diagnostic procedures and treatment.  In Table 1, it seems to me screening eligibility criteria should be compared using:  Number needed to be screened to prevent one lung cancer related death  Positive predictive value  Life years gained  Degree of disparities related to race/ethnicity, sex, socioeconomic status  Cost-effectiveness | Thank you. We have described our eligibility criteria for studies. It is uncertain whether screening should start at age 50, for example in younger people with elevated/high risk.   1. We will include RCTs with biomarkers if they report on one or more of our benefit outcomes. We agree that we do not expect to see any convincing evidence of this type of intervention yet (e.g. small if any trials). 2. We agree and have removed the word pre-screening. 3. In KQ3/Table 3 we are wanting to capture any differences in benefits and harms (rated as important/critical by the task force) from different interventions (based on their eligibility) and not focus on other outcomes such as detection rates, “false positive selections” etc. One may expect different rates of harms such as FPs or incidental findings with different populations selected for screening. Thank you for suggesting other outcomes to consider; there have been no changes to the outcomes listed in Table 1. |
| Marchuk | Yes | Will you be including conventional and low-dose CT scanning evidence? | Yes, we will include studies of conventional CT. We removed “low-dose” from the text describing our eligibility criteria |
| Norquay | No |  | Thank you. No changes requested |
| Pan | No |  | Thank you. No changes requested |
| Sanchez-Ramirez | No | The criteria are clearly presented in the tables and are well-aligned with the research questions. | Thank you. No changes requested |
| Sayani | Neither checked off | Specific populations of interest can include living with low income; precariously housed; homeless – all populations which have higher rates of smoking and poorer access.  For Q2 on values on preferences – qualitative studies can provide more richness to this understanding, particularly from the perspective of those currently underscreened – this is a limitation of the protocol design and eventual analysis which will miss this rich perspective and should be acknowledged | Thank you. To better account for sexual orientation as well as other specific populations requiring equity considerations we have revised our specific populations of interest (see Tables 1 and 2) to add “Populations for which screening access and outcomes may be inequitable (e.g., LGBTQ+, low socioeconomic status, homeless)” Smoking history is a major variable of interest for this review. Equity and accessibility are important considerations the Task Force considers using the review and other evidence/information such as stakeholder comments.  KQ2 currently focuses on quantitative assessment of the relative importance of the outcomes, to assist the task force when weighing the benefits and harms. We recognize that qualitative studies would be very useful if we were asked to examine patients experiences of cancer or barriers and facilitators of screening. The task force considers other input from patients when making their recommendations. In KQ2, studies examining preferences with use of decision aids etc may shed light on some of these factors. |
| Tracer | Yes | [For KQ3, The protocol does not specify which risk-prediction models will be evaluated. I assume it is all externally validated models. If so, do models need to be validated in Canadian populations? The protocol mentions PLCOm2012, which is based on a US cohort. Will applicability to Canadian populations be examined? PLCOm2012 (as an example) assigns higher risk of lung cancer based on Black race; this risk is likely based on social determinants. Will you examine whether this applies to the Canadian population? Conversely, risk prediction models calibrated and validated in non-Canadian populations likely won’t capture disparities in lung cancer relevant to Canada (e.g., lung cancer risk in Indigenous and First Nations people in Canada). For KQ1, modeling studies are excluded. The current CTFPHC recommendation appears to use the NLST as the source of eligibility criteria for screening (age, interval, duration of screening, pack-year smoking history). With the addition of a new trial (NELSON), the specific criteria for screening may be less clear. You might consider either commissioning modeling, or using already published modeling studies, to help inform the lung cancer screening recommendation. The applicability of existing modeling studies to the Canadian population would need to be examined. | Thank you for your input about the potential issues with applicability of some risk prediction models. Models will not be required to be validated in a Canadian population but this will be reported. We will take care when assessing the certainty of evidence (which considers applicability) with this in mind. KQ3 is likely to focus on published modeling studies and the task for is considering undertaking their own. |
| **Question 3: Do the research questions address the clinically important issues?** | | | |
| Bahig | Yes | Highly relevant and timely | Thank you. No changes requested |
| Boulanger | Yes | The questions asked are relevant in the present evaluation context and will confirm the relevance of screening as well as the different practices available. However, considering the clinical context and that some jurisdictions have started screening, additional questions could be addressed. For example, how long should the screening be? How many cycles should be done? | Thank you. The task force will consider this input for consideration when making recommendations. |
| Dennie | Yes |  | Thank you. No changes requested |
| Earle | No | More of a ‘yes, and…’: The duration of screening beyond that in the RCTs, i.e., not just annually for 3 years, is a very important question. The current Task Force recommendation doesn’t make real world sense in this regard IMO. | Thank you. The task force will consider this input for consideration when making recommendations. |
| Holmes | Yes | What are the benefits and harms of screening for x amount of years? The answer is likely part of question number one but it is important to determine how long screening should be continued for. Additionally, how do individuals at high risk for lung cancer weigh the benefits and harms of lung cancer screening, and how do they use this information in their decisions to undergo or not undergo screening? | Thank you. The task force will consider this input for consideration when making recommendations. The reviews at this time do not focus on how people make decisions on whether to screen. |
| Kulbak | Yes | What are the benefits and harms of screening for x amount of years? The answer is likely part of question number one but it is important to determine how long screening should be continued for. Additionally, how do individuals at high risk for lung cancer weigh the benefits and harms of lung cancer screening, and how do they use this information in their decisions to undergo or not undergo screening? | Thank you. The task force will consider this input for consideration when making recommendations. The reviews at this time do not focus on how people make decisions on whether to screen. |
| Lam | No | Not clearly. KQ3 a and b should be separate questions as they address different issues | Thank you. These questions are both about comparative effects between different screening strategies and will be reported separately. Oftentimes, questions on comparative effects include multiple types of comparisons. |
| Marchuk | Yes |  | Thank you. No changes requested |
| Norquay | Yes |  | Thank you. No changes requested |
| Pan | Yes | Key questions #1-3 appear adequately comprehensive to address clinically important issues. | Thank you. No changes requested |
| Sanchez-Ramirez | Yes | The research questions address important issues related to benefits, harms, values, and preferences around lung cancer screening. The results of this work will be valuable in informing future clinical guidelines and clinical practice. | Thank you. No changes requested |
| Sayani | Neither checked off | I would like to see us better set up to understand population level harms. Inequities can aggregate at a population-level from inequitable uptake of lung cancer screening in highest-risk population groups. This is important to guide resource allocation to support access to lung cancer screening. | Thank you. The task force will consider this input for consideration when making recommendations. Equity, accessibility, and resources are important additional considerations the Task Force considers using the review and other evidence/information such as stakeholder comments. |
| Tracer | Yes | Yes. Please see my comments under Q2 regarding the important issues of lung cancer risk in specific populations including Black persons and First Nations persons, and for questions regarding eligibility criteria for screening. | Thank you. |
| **Question 4: Are there any important sources of studies (i.e databases or organizational websites) that we did not include that should be considered in our review? If yes, please provide additional sources.** | | | |
| Bahig | No |  | Thank you. |
| Boulanger | No |  | Thank you. |
| Dennie | No |  | Thank you. |
| Earle | No | Not that I’m aware of | Thank you. |
| Holmes | No | N/A | Thank you. |
| Kulbak | No | N/A | Thank you. |
| Lam | Yes | I understand the scoping literature may be ongoing. I have attached publications that I think worthwhile to check if they are present in your search. Please see attached file | Thank you. We will screen these records. |
| Marchuk | No |  | Thank you. |
| Norquay | No |  | Thank you. |
| Pan | No |  | Thank you. |
| Sanchez-Ramirez | No | Not that I know | Thank you. |
| Sayani | Neither checked off | Sayani, A., Ali, M. A., Dey P., Corrado, A. M., Ziegler, C., Sadler, A., Williams, C., & Lofters, A. Interventions designed to increase the uptake of lung cancer screening: an equity-oriented scoping review. JTO Clinical and Research Report https://www.jtocrr.org/article/S2666-3643(23)00008-5/fulltext | Thank you. This does not seem eligible to our review questions but we will make the task force aware of this review for other considerations when making their recommendations. |
| Tracer | No |  |  |
| **Question 5: Are there specific reports or publications of research studies, or ongoing studies that might fit the inclusion criteria, that the Task Force should consider?** | | | |
| Bahig | No |  | Thank you. |
| Boulanger | No | A pilot project is currently underway in Quebec. The data is not published at this time. Planned end of evaluation: winter 2024. | Thank you. We will look for studies reporting results from pilots in Canada. |
| Dennie | No |  | Thank you. |
| Earle | No | Not that I’m aware of | Thank you. |
| Holmes | No | N/A | Thank you. |
| Kulbak | No | N/A | Thank you. |
| Lam | Yes | Please see attached file | Thank you. We will screen any research studies for eligibility for our review. |
| Marchuk | No |  | Thank you. |
| Norquay | No |  | Thank you. |
| Pan | No |  | Thank you. |
| Sanchez-Ramirez | No | Not that I know | Thank you. |
| Sayani | No |  | Thank you. |
| Tracer | No | Not that I am aware of. | Thank you. |
| **Question 6: Do you have any major concerns about the protocol that we should address?** | | | |
| Bahig | No | I wonder about the use of the future tense throughout the manuscript. | Our manuscript is for the protocol for describing the steps we will be taking in our reviews which had not yet started. |
| Boulanger | No |  | Thank you. |
| Dennie | No |  | Thank you. |
| Earle | No | No, aside from needing a duration of screening question as noted above. | Thank you. |
| Holmes | No | N/A | Thank you. |
| Kulbak | No | N/A | Thank you. |
| Lam | Yes | KQ3 a and b should be considered separately. ≥12 month follow up for false positive in Table 1 is too short. The standard clinical follow-up to determine benign status is 2 years for solid nodules and 5 years for subsolid nodules.A major issue is definition of a positive screen. Nodule management protocols need to be compared using quality indicators such as: Early reassessment rate (CT, PET); Invasive procedure rate (bronchoscopy, CT biopsy, surgery); Cancer detection rate per 1,000 people screened; Positive predictive values (of diagnostic work-up); Stage of screen detected lung cancers; Interval cancer rates; Lung cancer mortality rates; Health economic analysis. Incidental (or additional) findings on a screening CT is not necessary a “harm” but rather added health value for the participants. For example, detecting severe coronary artery calcification provide an opportunity for cardiac disease risk reduction (Please see: • Heuvelmans MA, Vonder M, Rook M, Groen HJM, De Bock GH, Xie X, Ijzerman MJ, Vliegenthart R, Oudkerk M. Screening for Early Lung Cancer, Chronic Obstructive Pulmonary Disease, and Cardiovascular Disease (the Big-3) Using Low-dose Chest Computed Tomography: Current Evidence and Technical Considerations. J Thorac Imaging 2019; 34: 160-169. •Ruparel M, Quaife SL, Dickson JL, Horst C, Tisi S, Hall H, Taylor MN, Ahmed A, Shaw PJ, Burke S, Soo MJ, Nair A, Devaraj A, Sennett K, Hurst JR, Duffy SW, Navani N, Bhowmik A, Baldwin DR, Janes SM. Prevalence, Symptom Burden, and Underdiagnosis of Chronic Obstructive Pulmonary Disease in a Lung Cancer Screening Cohort. Ann Am Thorac Soc 2020; 17: 869-878. •Klein-Awerianow. K RWOM, et al. Aortic stenosis as an additional finding in low-dose computed tomography lung cancer screening: a cross-sectional study. Ann Inter Med 2021; 174: 1482-1483.). •Pompe E, de Jong PA, Lynch DA, Lessmann N, Isgum I, van Ginneken B, Lammers JJ, Mohamed Hoesein FAA. Computed tomographic findings in subjects who died from respiratory disease in the National Lung Screening Trial. Eur Respir J. 2017 Apr 19;49(4):1601814. doi: 10.1183/13993003.01814-2016. Print 2017 Apr. PMID: 28424361 | Thank you.  Kindly review our response about KQ3 which we recognize looks at 2 different comparisons but will be reported separately.  We recognize that more than 12 months may be required to resolve positive screening exams. This timeframe is a minimum for eligibility though we may consider studies with this timeframe as possibly not capturing accurate numbers. The research studies may allow for quicker resolution than seen in typical screening programs. We will make sure we are aware of who is counted in the outcome reporting e.g. ideally only those who have had complete diagnostic workup, and the amount of missing data because of this. We have added comments about this to the protocol.  We will report the definition of a positive screen for each study but are interested in final benign diagnosis after recall for additional imaging and/or for full diagnostic workup.  The task force has not identified cancer detection, PPV, stage of disease, interval cancers to be critical outcomes of interest for their decision making. Cost-effectiveness analysis may be undertaken or considered (eg others’ analyses) during the development of recommendations but is not part of this set of reviews.  We recognize that incidental findings will not all be harmful, though patient-relevant outcomes with respect of these will not be able to be directly assessed in the studies; the task force also recognizes that incidental findings may also have resource and other considerations for primary care providers. We will delineate as best possible between “clinically significant” and “any IF” outcomes, and to also perform analysis on certain specific IFs of interest, to help with this interpretation. We will share these papers with the task force and screen them for eligibility in our review. |
| Marchuk | No | I have a question about the following statement as I am not sure what is meant by FP, I am not sure if you are referring the family physician, if so I would ask that we expand this to include NPs as well. “No limitation on the duration of follow-up, except for FPs where ≥12 mos follow-up after the screening result is required in ≥80% of participants (if there is no evidence found meeting this criteria we will accept ≥6 mos follow-up). | Thank you. FPs refers to the false positive outcome. NPs are definitely considered primary care providers by the task force. |
| Norquay | No |  | Thank you. |
| Pan | No | I think the protocol addresses several important questions for the Task Force, and it is important to keep this updated as new evidence emerges. | Thank you. |
| Sanchez-Ramirez | No | This protocol is well-developed. It supports the importance of this work and provides detailed information on the decision process related to the eligibility criteria and on the intended approach. | Thank you. |
| Sayani | Neither checked off | It is important that the patient stakeholders who inform the equity perspectives of the reviews have lived/ living experiences of social inequity. | Thank you. We will share this input with the task force and knowledge translation group undertaking this work for the task force. |
| Tracer | No |  | Thank you. |
| Other comments | | | |
| Manthorne  Canadian Cancer Survivor Network | Via e-mail  I have read through the review document and have a few observations.  First, have you given any thought to collecting data from lung cancer patients, caregivers, and  survivors about their experiences with lung cancer? The lung cancer community in Canada is  very active, with several support groups as well as a Right2Survive coalition, with the  Canadian Cancer Survivor Network lead organization, and Lung Cancer Canada and the Lung  Health Foundation as partners. This coalition has a website - right2survive.ca, and a mailing  list of approximately 2,400 subscribers to a quarterly newsletter. Recognizing real world  evidence about the lived experiences of those diagnosed with and living with lung cancer has  been recognized by CADTH and INESSS as well as several provincial drug approval bodies  as an appropriate method of understanding the patient experience.  In addition, I would like to ask the Task Force to consider not putting an upper age cutoff for  lung cancer screening to recognize that lung cancer (like most cancers) is diagnosed more  often as people age. Cutoff dates make it difficult if not impossible for people at risk to be  screened. | | Thank you for your comments. The task force seeks input from patient partners (usually with and without experience of the disease) during the development of recommendations. This additional work supplements findings from these reviews.  We will share your input on stopping age with the task force. |
| Julie Lessard, Institut national d’excellence en sante et en services sociaux (INESSS) | Comments in protocol manuscript   1. Add “protocol” to title 2. Abstract: This citation from the discussion could appear earlier as it helps to understand the work that will be done and its role in Task force recommendation. “The results will be used by the task force for developing recommendations about screening for lung cancer with low-dose CT. It will also serve as a comprehensive review for clinicians and other decision makers on the effects of screening and relevant patient preferences.” Also, in the Background section, there could be a small section on the Task force works – 3. Re background on costs per case - interesting to mention which costs? new expensive drugs? 4. Re harms amongst those with a FP - any statistics about these ones and overdiagnosis? 5. Re NLST positive findings including those with incidental findings - including benign findings? 6. Sentence “apart from nodule classification, there …different selection criteria - nodule classification systems are not use to refine patient selection criteria for screening…., is a way to report screening result, I don’t understand the link made here as nodule classification is done after screening selection 7. Scope and purpose: - may be this could be at the beginning of the background section, it will help to understand what will be do, and why 8. KQ2 wording - is this a literature review or input from patient performing consultation? | | Thanks very much.   1. We added protocol to the title thanks for catching this. 2. We have also been asked to add some background to the abstract, hence did not add any other comments; the background section has some information on how the task force will use the review but we added the sentence about use of the data for other audiences at the end of the background section. 3. We looked closely and could not figure out where this estimate from the Statistics Canada report came from r what cost were included. We have revised based on a detailed report in Alberta. 4. We did not find any statistics about these harms since our reviews will look at those. 5. The NSLT positive screening test included other incidental findings (eg cardiovascular) which are often not potentially cancerous 6. We have revised the sentence to remove the comparison with nodule classification when discussing selection criteria. 7. We have added a citation to the task force methods manual. 8. This is our review, the task force will use other input as well |
| Boulanger | Comments in protocol manuscript   1. Additional statistics to consider 2. Chemotherapy for stage II - Targeted anti-EGFR therapy (osimertinib) or immunotherapy (atezolizumab) could also be considered. 3. SCLC treatment - Atezolizumab or durvalumab can also be used for extended stages 4. Screening aims – effectiveness- ideally curative and not palliative | | 1. Thank you, we have added some updated stats 2. We added this 3. We added immunotherapies as a possibility 4. We revised to curative |
| Vella | Comments in protocol manuscript   1. Include “protocol” in title 2. Data extraction items - You might want to extract the factors that were controlled for in the non-RCTs. 3. You might want to list the potential confounders in your tables. 4. Risk of bias - Will you be using the QUADAS-2 tool to assess ROB of test accuracy outcomes? Will you be using the PROBAST tool to assess the ROB of prediction models? 5. Certainty assessments for psychosocial outcomes - This seems to indicate that you want comparative studies between screening vs. no screening for psychosocial outcomes. 6. Table 1 harms outcomes - Would some of these harms come from the RCTs? Also, could you include comparative (between different screening strategies) nonrandomized studies for these harms? 7. Table 1 What about comparative nonrandomized studies of screening strategies where both strategies were used in RCTs in KQ1? Can you include harms from these studies in KQ1 or are you only including noncomparative studies for harms for KQ1? | | Thank you   1. Thanks for catching this error; we’ve added 2. We added that for nonRCTs (used for harms only in KQ1) we will extract details of an adjusted analyses for nonRCTs, 3. We have outlined our main confounders of interest 4. Our outcome of false positives is the number of people with one or more vs. accuracy data like FP rates. We do not have any other accuracy outcomes of interest. We are not accessing the performance of risk prediction models but rather using data on outcomes from implementation of these in practice (on patient-important outcomes). 5. Correct, we will include uncontrolled studies but assess those with comparisons at higher certainty (due to less risk of bias). We have clarified that RCTs will be the only evidence to start at high certainty for these outcomes though. 6. We will definitely include harms from RCTs, but also include nonRCTs for these outcomes; we clarified this in the text on p. 14 7. For KQ1, harms may be included from any study as long as one or more of the strategies is similar to one of the RCTs with a usual care arm. So for an RCT comparing 2 strategies we would extract the harms from each eligible arm. |
| Lam | Comments in protocol manuscript  Most mentioned above except:   1. Is pack-years sufficient? 2. Is contamination data available? 3. KQ3 table 3: i) Risk prediction models to determine screening eligibility should be separated from risk prediction models for nodule assessment, ii) exclusion of RCTs -why? | | Thank you.   1. We will collect any reported data on smoking history. 2. We are uncertain about how much data on contamination will be available and may need to adjust this criterion (and report this) if there is very poor reporting on this. 3. KQ3a is looking at selection criteria (with a focus on RPMs vs risk-based criteria as used in most RCTs) and KQ3b looks at different nodule classification (of which some may use RPMs). We have clarified that RPMs incorporating nodule characteristics will only be excluded for KQ3a. The purpose of KQ3 is to take a close look at observational studies and modelling studies on comparative effects of different strategies; KQ1 will also include these comparisons in RCTs, if they exist. |
