# Supplementary material for: Screening for lung cancer with computed tomography: protocol for systematic reviews for the Canadian Task Force on Preventive Health Care
Source: Syst Rev. 2024 Mar 16;13:88. doi: 10.1186/s13643-024-02506-3 (PMC10943889; doi:10.1186/s13643-024-02506-3)
Supplement: Supplementary file 2 — Additional file 2. MEDLINE Search Strategies. [file 13643_2024_2506_MOESM2_ESM.docx]

**Screening for Lung Cancer with Computed Tomography: Systematic Reviews for the Canadian Task Force on Preventive Health Care**

**Supplementary file 2**

**MEDLINE Search Strategies**

**Key Question 1**

Database(s): **Ovid MEDLINE(R) ALL**1946 to October 05, 2022

| **#** | **Searches** | **Results** | **Annotations** |
| --- | --- | --- | --- |
| 1 | exp Lung Neoplasms/ | 265295 |  |
| 2 | ((bronchial or lung or pulmonary) adj3 (adenocarcinoma or cancer* or carcinoma or malignan* or neoplasm* or tumo?r*)).ti,ab,kf. | 272155 |  |
| 3 | 1 or 2 | 358120 | Lung CA |
| 4 | Early Detection of Cancer/ | 35089 |  |
| 5 | Mass Screening/ | 114684 |  |
| 6 | Tomography, X-Ray Computed/ | 412000 |  |
| 7 | (compute* adj2 tomograph*).ti,ab,kf. | 366992 |  |
| 8 | (c?t adj1 (chest* or imag* or scan*)).ti,ab,kf. | 157012 |  |
| 9 | (LDCT or LD-CT or ULDCT or ULD-CT).ti,ab,kf. | 1431 |  |
| 10 | (low-dose adj ct*).ti,ab,kf. | 2682 |  |
| 11 | screen*.ti,ab,kf. | 915416 |  |
| 12 | or/4-11 | 1608060 | Screening |
| 13 | 3 and 12 | 51016 | Lung CA + Screening |
| 14 | limit 13 to yr="2015 -Current" | 23847 | Lung CA + Mass Screening/CT: 2015-Current |
| 15 | exp Radiography, Thoracic/ | 40510 |  |
| 16 | Sputum/cy [Cytology] | 3054 |  |
| 17 | ((chest or thoracic or thorax) adj1 (radiograph* or xray* or x-ray*)).ti,ab,kf. | 54899 |  |
| 18 | (sputum adj2 cyto*).ti,ab,kf. | 1055 |  |
| 19 | or/15-18 | 85308 |  |
| 20 | 3 and 19 | 10630 | Lung CA + CXR/sputum |
| 21 | limit 20 to yr="2020 -Current" | 519 | Lung CA + CXR/sputum: 2020-current |
| 22 | 14 or 21 | 24004 | Lung CA + Screening |
| 23 | Randomization/ | 106884 |  |
| 24 | Random Allocation/ | 106884 |  |
| 25 | Double-Blind Method/ | 173167 |  |
| 26 | Double-Blind Studies/ | 173167 |  |
| 27 | Single-Blind Method/ | 32209 |  |
| 28 | Single-Blind Studies/ | 32209 |  |
| 29 | Control Groups/ | 1860 |  |
| 30 | allocated.ti,ab,hw. | 79429 |  |
| 31 | (control* adj3 (group* or studies or study or trial*)).ti,ab,kf. | 1166254 |  |
| 32 | ((Danish or Dante or German or LUSI or MILD or Nelson or UK) adj5 (lung* or trial)).ti,ab. | 7355 |  |
| 33 | (DLCST or DLST or Italung or NLST or UKLS).ti,ab. | 738 |  |
| 34 | ((doubl* or singl* or trebl* or triple) adj (blind* or dumm* or mask*)).ti,ab,hw,kf. | 261914 |  |
| 35 | random*.ti,ab,hw,kf. | 1601910 |  |
| 36 | or/23-35 | 2302902 | RCT filter |
| 37 | 22 and 36 | 2388 | LungCA screening RCTs |
| 38 | exp Lung Neoplasms/ | 265295 |  |
| 39 | ((bronchial or lung or pulmonary) adj3 (adenocarcinoma or cancer* or carcinoma or malignan* or neoplasm* or tumo?r*)).ti,ab,kf. | 272155 |  |
| 40 | 38 or 39 | 358120 | Lung CA |
| 41 | Early Detection of Cancer/ | 35089 |  |
| 42 | Mass Screening/ | 114684 |  |
| 43 | Tomography, X-Ray Computed/ | 412000 |  |
| 44 | (compute* adj2 tomograph*).ti,ab,kf. | 366992 |  |
| 45 | (c?t adj1 (chest* or imag* or scan*)).ti,ab,kf. | 157012 |  |
| 46 | (LDCT or LD-CT or ULDCT or ULD-CT).ti,ab,kf. | 1431 |  |
| 47 | (low-dose adj ct*).ti,ab,kf. | 2682 |  |
| 48 | screen*.ti,ab,kf. | 915416 |  |
| 49 | or/41-48 | 1608060 | Screening |
| 50 | 40 and 49 | 51016 | Lung CA + Screening |
| 51 | limit 50 to yr="2015 -Current" | 23847 | Lung CA + Mass Screening/CT: 2015-Current |
| 52 | exp Radiography, Thoracic/ | 40510 |  |
| 53 | Sputum/cy [Cytology] | 3054 |  |
| 54 | ((chest or thoracic or thorax) adj1 (radiograph* or xray* or x-ray*)).ti,ab,kf. | 54899 |  |
| 55 | (sputum adj2 cyto*).ti,ab,kf. | 1055 |  |
| 56 | or/52-55 | 85308 |  |
| 57 | 40 and 56 | 10630 | Lung CA + CXR/sputum |
| 58 | limit 57 to yr="2020 -Current" | 519 | Lung CA + CXR/sputum: 2020-current |
| 59 | 51 or 58 | 24004 | Lung CA + Screening |
| 60 | exp Anti-Anxiety Agents/ | 73006 |  |
| 61 | exp Antidepressive Agents/ | 157968 |  |
| 62 | Anxiety/ | 100991 |  |
| 63 | denial, psychological/ | 2655 |  |
| 64 | Depression/ | 144179 |  |
| 65 | exp Fear/ | 38335 |  |
| 66 | Mental Health/ | 55872 |  |
| 67 | Psychological Distress/ | 3569 |  |
| 68 | Psychometrics/ | 86086 |  |
| 69 | exp Psychiatric Status Rating Scales/ | 86867 |  |
| 70 | Stress, Psychological/ | 131872 |  |
| 71 | (afraid or fear*).ti,ab,kf. | 103531 |  |
| 72 | (antianxiety or anti-anxiety or anxiolytic* or tranquiliz*).ti,ab,kf. | 25334 |  |
| 73 | (antidepress* or anti-depress*).ti,ab,kf. | 77877 |  |
| 74 | (anxiet* or anxious*).ti,ab,kf. | 254673 |  |
| 75 | BDI-II.ti,ab,kf. | 2547 |  |
| 76 | ((cancer or worry) adj2 scale*).ti,ab,kf. | 1854 |  |
| 77 | (consequence*-of-screening* or COS or COS-LC).ti,ab,kf. | 19546 |  |
| 78 | (denial or deny).ti,ab,kf. | 10734 |  |
| 79 | depressi*.ti,ab,kf. | 463445 |  |
| 80 | distress*.ti,ab,kf. | 156226 |  |
| 81 | ((emotion* or psychological or psychosocial or psycho-social) adj2 (aftereffect* or aspect* or burden* or consequence* or discomfort* or effect* or impact* or measure* or outcome* or reaction* or response* or symptom*)).ti,ab,kf. | 105779 |  |
| 82 | HADS.ti,ab,kf. | 7090 |  |
| 83 | (impact-of-event*-scale* or IES).ti,ab,kf. | 4602 |  |
| 84 | mental-health.ti,ab,kf. | 205797 |  |
| 85 | (patient*-health-questionnaire* or PHQ9 or PHQ-9).ti,ab,kf. | 10702 |  |
| 86 | PCQ.ti,ab,kf. | 243 |  |
| 87 | ((psychological or psychosocial or psycho-social) adj2 (assess* or questionnaire* or scale* or screen* or test*)).ti,ab,kf. | 25135 |  |
| 88 | (psychometr* or psycho-metr*).ti,ab,kf. | 59651 |  |
| 89 | STAI.ti,ab,kf. | 4267 |  |
| 90 | stress*.ti,ab,kf. | 1027154 |  |
| 91 | or/60-90 | 2273792 | Psychological harms |
| 92 | Diagnostic Errors/ | 39469 |  |
| 93 | False Positive Reactions/ | 28578 |  |
| 94 | Incidental Findings/ | 11621 |  |
| 95 | exp Medical Overuse/ | 14616 |  |
| 96 | ((adverse or harm* or serious or undesir* or uninten* or unwant*) adj3 (effect* or event* or outcome* or reaction*)).ti,ab,kf. | 611284 |  |
| 97 | ((bleed* or complication* or harm* or infection*) adj3 (biops* or death* or followup* or follow-up* or morbidity or mortality or procedure* or resect* or surg* or test* or treatment* or workup* or work-up*)).ti,ab,kf. | 287421 |  |
| 98 | ((extrapulmonary or extra-pulmonary or incidental) adj3 (discover* or finding*)).ti,ab,kf. | 13306 |  |
| 99 | false*-positiv*.ti,ab,kf. | 67617 |  |
| 100 | (invasive adj2 (follow-up* or followup* or procedure* or test* or treatment* or workup* or work up*)).ti,ab,kf. | 50681 |  |
| 101 | (overdetect* or over-detect* or overdiagnos* or over-diagnos* or overtreat* or over-treat*).ti,ab,kf. | 14254 |  |
| 102 | (unnecessary adj2 (biops* or followup* or follow-up* or procedur* or resect* or surg* or test* or treatment* or workup* or work-up*)).ti,ab,kf. | 13608 |  |
| 103 | or/92-102 | 1096062 | Other harms |
| 104 | 59 and (91 or 103) | 3119 | Lung CA Screening + (Psychological or Other Harms) |
| 105 | Epidemiologic studies/ | 9175 |  |
| 106 | exp case control studies/ | 1358431 |  |
| 107 | exp cohort studies/ | 2401237 |  |
| 108 | Case control.tw. | 146915 |  |
| 109 | (cohort adj (study or studies)).tw. | 286941 |  |
| 110 | Cohort analy$.tw. | 10808 |  |
| 111 | ((Follow-up or followup) adj (study or studies)).tw. | 55246 |  |
| 112 | (observational adj (study or studies)).tw. | 146891 |  |
| 113 | Longitudinal.tw. | 302027 |  |
| 114 | Retrospective.tw. | 689162 |  |
| 115 | (Cross-sectional or crosssectional).tw. | 470959 |  |
| 116 | Cross-sectional studies/ | 441950 |  |
| 117 | ((control and (group* or study)) or (time and factors) or program or survey* or ci or cohort or comparative-stud* or evaluation-studies or follow-up*).mp. | 8288809 |  |
| 118 | or/105-117 | 9282176 | Non-RCT study designs |
| 119 | 104 and 118 | 1637 | LungCA Screening Harms non-RCTs |
| 120 | 37 or 119 | 3667 | LungCA Screening Benefits or Harms |
| 121 | (Case Reports.pt. or (case report? or case study or case studies).ti.) not (review* or trial*).ti,ab,kf,hw. | 2095372 |  |
| 122 | comment/ or editorial/ or (comment or editorial or news or newspaper article).pt. | 1621308 |  |
| 123 | 120 not (121 or 122) | 3553 |  |
| 124 | remove duplicates from 123 | 3546 |  |

**Key Question 2**

Database(s): Ovid MEDLINE(R) ALL 1946 to November 15, 2022

| **#** | **Searches** | **Results** | **Annotations** |
| --- | --- | --- | --- |
| 1 | exp Lung Neoplasms/ | 266867 |  |
| 2 | ((bronchial or lung or pulmonary) adj3 (adenocarcinoma or cancer* or carcinoma or lesion* or malignan* or mass$2 or neoplasm* or tumo?r* or nodule*)).ti,ab,kf. | 299233 |  |
| 3 | 1 or 2 | 379337 |  |
| 4 | Early Detection of Cancer/ | 35503 |  |
| 5 | Mass Screening/ | 115034 |  |
| 6 | (screen* or detect*).ti,ab,kf. | 3409436 |  |
| 7 | or/4-6 | 3440454 |  |
| 8 | 3 and 7 | 62831 | LungCA Screening |
| 9 | attitude to health/ | 85380 |  |
| 10 | Choice Behavior/ | 34671 |  |
| 11 | Consumer Behavior/ | 23656 |  |
| 12 | Decision Making/ | 103521 |  |
| 13 | Decision Making, Shared/ | 1736 |  |
| 14 | decision support techniques/ | 22341 |  |
| 15 | health knowledge, attitudes, practice/ | 125001 |  |
| 16 | "patient acceptance of health care"/ | 54010 |  |
| 17 | patient participation/ | 29009 |  |
| 18 | patient preference/ | 10455 |  |
| 19 | patient satisfaction/ | 88529 |  |
| 20 | Perception/ | 42305 |  |
| 21 | ((acceptabilit* or acceptance or acceptable or attitude* or belief* or expectation* or intent* or perceiv* or perception* or perspective* or prefer* or valuation* or valued or values or views or willing*) adj4 (elicit* or client* or consumer* or female* or health or individual* or man or men or male$1 or participant* or patient* or people* or person* or public* or respondent* or screenee* or smoker* or stated or user* or wom#n)).ti,ab,kf. | 465139 |  |
| 22 | ((choice* or choos*) adj2 (behavio?r* or discrete or experiment*)).ti,ab,kf. | 9474 |  |
| 23 | (conjoint adj3 (analy* or design* or evaluation or exercise* or experiment* or studies or study or survey* or task* or valuation or value* or valuing)).ti,ab,kf. | 1352 |  |
| 24 | (contingent adj3 (analy* or design* or evaluation or valuation or value* or valuing)).ti,ab,kf. | 1125 |  |
| 25 | (decision* adj (board$1 or certain* or conflict* or dissatisf* or satisf* or uncertain*)).ti,ab,kf. | 2082 |  |
| 26 | (decision* adj2 (aid$1 or mak* or needs or support* or tool*)).ti,ab,kf. | 244821 |  |
| 27 | (decision* adj5 balance).ti,ab,kf. | 1152 |  |
| 28 | (ranking or rating).ti,ab,kf. | 152035 |  |
| 29 | (("LCS" or screen*) adj4 (acceptabilit*or acceptance or acceptable or agree* or attitude* or behavio?r* or belief* or choice* or choos* or decid* or decision* or declin* or expectation* or inclin* or intending or intent* or perceiv* or perception* or perspective* or prefer*or propensity or pursue or values or views or willing*)).ti,ab,kf. | 20643 |  |
| 30 | (tradeoff* or trade-off*).ti,ab,kf. | 38560 |  |
| 31 | or/9-30 | 1253550 | Patient Preferences |
| 32 | 8 and 31 | 2231 | LungCA Screening + Patient preferences |
| 33 | (Case Reports.pt. or (case report? or case study or case studies).ti.) not (review* or trial*).ti,ab,kf,hw. | 2102023 |  |
| 34 | comment/ or editorial/ or (comment or editorial or news or newspaper article).pt. | 1630083 |  |
| 35 | (exp Animals/ or Models, Animal/ or Disease Models, Animal/) not Humans/ | 5065889 |  |
| 36 | ((animal or animals or canine* or dog or dogs or feline or hamster* or lamb or lambs or mice or monkey or monkeys or mouse or murine or pig or pigs or piglet* or porcine or primate* or rabbit* or rats or rat or rodent* or sheep* or veterinar*) not (human* or patient*)).mp. | 4930092 |  |
| 37 | 32 not (or/33-36) | 2137 |  |
| 38 | limit 37 to yr="2012-Current" | 1596 |  |
| 39 | remove duplicates from 38 | 1591 | LungCA Screening Acceptability/Preferences 2012-Current |
| 40 | exp Lung Neoplasms/ | 266867 |  |
| 41 | ((bronchial or bronchus or lung or pulmonary) adj3 (adenocarcinoma or cancer* or carcinoma or malignan* or neoplasm* or tumo?r*)).ti,ab,kf. | 275198 |  |
| 42 | 40 or 41 | 360777 | LungCA |
| 43 | Diagnostic Errors/ | 39514 |  |
| 44 | False Positive Reactions/ | 28579 |  |
| 45 | Incidental Findings/ | 11643 |  |
| 46 | Precancerous Conditions/ | 29297 |  |
| 47 | Solitary Pulmonary Nodule/ | 4601 |  |
| 48 | ((bronchial or bronchus or lung or pulmonary) adj3 (lesion* or mass$2 or node* or nodule*)).ti,ab,kf. | 40891 |  |
| 49 | ((extrapulmonary or extra-pulmonary or incidental) adj3 (discover* or finding*)).ti,ab,kf. | 13399 |  |
| 50 | (false*-positiv* or overdiagnosis or (diagnos* adj3 error*)).ti,ab,kf. | 79280 |  |
| 51 | (indetermina* or suspect* or suspicious*).ti,ab,kf. | 290643 |  |
| 52 | or/43-51 | 493608 |  |
| 53 | (bronchial or bronchus or lung or pulmonary).hw,ti,ab,kf. | 1440868 |  |
| 54 | 52 and 53 | 76898 | Lung non-CA |
| 55 | Quality-Adjusted Life Years/ | 15220 |  |
| 56 | *"Quality of Life"/ | 109052 |  |
| 57 | "Value of Life"/ | 5793 |  |
| 58 | Visual Analog Scale/ | 4078 |  |
| 59 | (DALY or DALYS or QALY or QALYS).ti,ab,kf. | 17622 |  |
| 60 | direct*-elicitation*.ti,ab,kf. | 51 |  |
| 61 | disabil*-weight*.ti,ab,kf. | 444 |  |
| 62 | ((disability or quality) adj adjusted-life).ti,ab,kf. | 21119 |  |
| 63 | eortc-qlq-30c.ti,ab,kf. | 2 |  |
| 64 | (EuroQol5D or EuroQol or Euro-Qol or EuroQual5D or EuroQual or Euro-Qual or EQ5D or EQ-5D).ti,ab,kf. | 15415 |  |
| 65 | fact-i.ti,ab,kf. | 50 |  |
| 66 | (health adj2 (utilit* or disutilit*)).ti,ab,kf. | 3986 |  |
| 67 | (health* adj2 year* adj2 equivalent*).ti,ab,kf. | 48 |  |
| 68 | (health-stat$2 adj2 (cost* or estimat* or index or indice* or measure* or scale* or score* or valu* or weight*)).ti,ab,kf. | 6405 |  |
| 69 | ((HRQOL or QOL or quality-of-life) adj3 (assess* or instrument*)).ti,ab,kf. | 34321 |  |
| 70 | (HSUV or HUSVs).ti,ab,kf. | 48 |  |
| 71 | (HUI or HUI2 or HUI3).ti,ab,kf. | 1868 |  |
| 72 | (HYE or HYES).ti,ab,kf. | 75 |  |
| 73 | (multi-attribute or multi-criteria or multiattribute or multicriteria).tw,kf. | 4289 |  |
| 74 | (quality adj2 (wellbeing or well-being)).ti,ab,kf. | 2604 |  |
| 75 | (sf6 or sf-6 or short-form-6 or shortform-6 or sf-six or sfsix or shortform-six or short-form-six or shortform6 or short-form6).ti,ab,kf. | 2526 |  |
| 76 | (sf8 or sf-8 or sf-eight or sfeight or shortform-8 or shortform-8 or shortform8 or short-form8 or shortform-eight or short-form-eight).ti,ab,kf. | 596 |  |
| 77 | (sf12 or sf-12 or short-form-12 or shortform-12 or short-form12 or shortform12 or sf-twelve or sftwelve or shortform-twelve or short-form-twelve).ti,ab,kf. | 7298 |  |
| 78 | (sf16 or sf-16 or short-form-16 or shortform-16 or short-form16 or shortform16 or sf-sixteen or sfsixteen or shortform-sixteen or short-form-sixteen).ti,ab,kf. | 39 |  |
| 79 | (sf20 or sf-20 or short-form-20 or shortform-20 or short-form20 or shortform20 or sf-twenty or sftwenty or shortform-twenty or short-form-twenty).ti,ab,kf. | 445 |  |
| 80 | (sf36 or sf-36 or short-form-36 or shortform-36 or short-form36 or shortform36 or sf-thirtysix or sfthirtysix or sfthirty-six or sf-thirty-six or shortform-thirtysix or shortform-thirty-six or short-form-thirtysix or short-form-thirty-six).ti,ab,kf. | 29670 |  |
| 81 | standard gamble*.ti,ab,kf. | 904 |  |
| 82 | (time adj (tradeoff or trade-off)).ti,ab,kf. | 1602 |  |
| 83 | TTO.ti,ab,kf. | 1321 |  |
| 84 | (utility adj (cost* or estimat* or index or indice* or measure* or scale* or score* or valu* or weight*)).ti,ab,kf. | 5550 |  |
| 85 | vignette*.ti,ab,kf. | 13244 |  |
| 86 | visual-analog*-scale*.ti,ab,kf. | 69429 |  |
| 87 | or/55-86 | 272469 | HSUV |
| 88 | 42 and 87 | 3176 | LungCA + HSUV |
| 89 | 54 and 87 | 265 | Lung non-CA + HSUV |
| 90 | 88 or 89 | 3320 |  |
| 91 | (Case Reports.pt. or (case report? or case study or case studies).ti.) not (review* or trial*).ti,ab,kf,hw. | 2102023 |  |
| 92 | comment/ or editorial/ or (comment or editorial or news or newspaper article).pt. | 1630083 |  |
| 93 | (exp Animals/ or Models, Animal/ or Disease Models, Animal/) not Humans/ | 5065889 |  |
| 94 | ((animal or animals or canine* or dog or dogs or feline or hamster* or lamb or lambs or mice or monkey or monkeys or mouse or murine or pig or pigs or piglet* or porcine or primate* or rabbit* or rats or rat or rodent* or sheep* or veterinar*) not (human* or patient*)).mp. | 4930092 |  |
| 95 | 90 not (or/91-94) | 3171 |  |
| 96 | limit 95 to yr="2012 -Current" | 2079 |  |
| 97 | remove duplicates from 96 | 2072 |  |
| 98 | 39 or 97 | 3593 | LungCA Screening preferences/acceptability OR LungCA HSUV |

**Key Question 3**

**Database(s):** Ovid MEDLINE(R) ALL 1946 to December 02, 2022

| **#** | **Searches** | **Results** | **Annotations** |
| --- | --- | --- | --- |
| 1 | exp Lung Neoplasms/ | 267326 |  |
| 2 | ((bronchial or lung or pulmonary) adj3 (adenocarcinoma or cancer* or carcinoma or lesion* or malignan* or mass$2 or neoplasm* or tumo?r* or nodule*)).ti,ab,kf. | 300068 |  |
| 3 | 1 or 2 | 380247 | LungCA |
| 4 | Early Detection of Cancer/ | 35688 |  |
| 5 | Mass Screening/ | 115160 |  |
| 6 | (screen* or detect*).ti,ab,kf. | 3418807 |  |
| 7 | or/4-6 | 3449838 | Screening |
| 8 | 3 and 7 | 63012 | LungCA Screening |
| 9 | ((nodule adj3 threshold*) or (nodule adj3 evaluat*) or (nodule adj3 classifi*) or (nodule adj3 categor*)).ti,ab,kf. | 1029 |  |
| 10 | (BIMC or Bayesian Inference Malignancy Calculator or "Bayesian Method" or "Brock University cancer prediction equation" or "Lung Nodule Malignancy Risk Calculator" or "McWilliams nodule malignancy score for risk prediction" or "Solitary Pulmonary Nodule Malignancy Risk Score" or "Mayo Clinic Model" or "UKLS Nodule Risk" or UKLS-NRM or uklsnrm or ("UK Lung Screen" adj3 model) or ((BTS or British Thoracic Society) adj4 nodule)).ti,ab,kf. | 2025 |  |
| 11 | ((Brock or Herder or Mayo or McWilliams or Pan-CAN or PanCAN or Pan-Canadian Early Detection of Lung Cancer or "Veterans Administration") adj3 (calculat* or score* or risk* or tool or model)).ti,ab,kf. | 3653 |  |
| 12 | ("Lung cancer risk assessment tool" or "Lung cancer death risk assessment tool" or LCRAT or LCDRAT or "multiscale lung nodule prediction strategy" or "Vancouver Risk Calculator" or "BTS guidelines" or "British Thoracic Society guidelines").ti,ab,kf. | 253 |  |
| 13 | ("Lung CT Screening Reporting and Data System" or lung-RADs or lungRADS or I-ELCAP or NODCAT or "Pulmonary Nodule Plasma Proteomic Classifier").ti,ab,kf. | 231 |  |
| 14 | or/9-13 | 7085 |  |
| 15 | limit 14 to yr="2012 -Current" | 5086 |  |
| 16 | exp "Predictive Value of Tests"/ | 221854 |  |
| 17 | exp Risk/ | 1360343 |  |
| 18 | exp "Sensitivity and Specificity"/ | 642042 |  |
| 19 | assessment-tool$1.ti,ab. | 33458 |  |
| 20 | (micro-simulation* or microsimulation).ti,ab. | 1832 |  |
| 21 | (predict* adj3 (clinical or model* or outcome* or rule$1 or scor*)).ti,ab. | 327299 |  |
| 22 | (risk adj3 (assessment$1 or clinical or death or model$1 or mortality or predict* or target*)).ti,ab. | 331455 |  |
| 23 | simulation-model$1.ti,ab. | 11540 |  |
| 24 | Statistical model$1.ti,ab. | 16076 |  |
| 25 | ((Bach or Hoggart or "Hunt Lung Cancer" or Kovalchik or Knoke or "Liverpool lung project risk" or LLP or "UKLS Nodule Risk" or Spitz or Mookgavkar or "Moolgavkar and Knudson" or "Two-Stage Clonal Expansion" or TSCE) adj3 (model or score)).ti,ab. | 131 |  |
| 26 | (PLCO adj3 (calculator or model or score or tool)).ti,ab. | 10 |  |
| 27 | or/16-26 | 2357006 |  |
| 28 | limit 27 to yr="2019-Current" | 465470 | Other RPM: 2019-current |
| 29 | 15 or 28 | 470066 | Nodule 2012-current OR Other RPM 2019-current |
| 30 | 8 and 29 | 2916 | LungCA screening + (nodule or RPM) |
| 31 | Epidemiologic studies/ | 9209 |  |
| 32 | exp case control studies/ | 1373066 |  |
| 33 | exp cohort studies/ | 2422156 |  |
| 34 | Case control.tw. | 148431 |  |
| 35 | (cohort adj (study or studies)).tw. | 293201 |  |
| 36 | Cohort analy$.tw. | 10992 |  |
| 37 | ((Follow-up or followup) adj (study or studies)).tw. | 55650 |  |
| 38 | (observational adj (study or studies)).tw. | 149927 |  |
| 39 | Longitudinal.tw. | 306375 |  |
| 40 | Retrospective.tw. | 700569 |  |
| 41 | (Cross-sectional or crosssectional).tw. | 480040 |  |
| 42 | Cross-sectional studies/ | 448611 |  |
| 43 | ((control and (group* or study)) or (time and factors) or program or survey* or ci or cohort or comparative-stud* or evaluation-studies or follow-up*).mp. | 8355037 |  |
| 44 | or/31-43 | 9361243 | Non-RCT study designs |
| 45 | exp Validation Study/ | 109092 |  |
| 46 | exp "Sensitivity and Specificity"/ | 642042 |  |
| 47 | (validat* or validity or sensitiv* or specific* or reliability or reliable).ti,ab,kf. | 5598879 |  |
| 48 | or/45-47 | 5904145 | Validation studies |
| 49 | 44 or 48 | 13165133 | Non-RCTs or validation studies |
| 50 | 30 and 49 | 2522 | LungCA Screening + (nodule 2012-current OR other RPM 2019-current) + Study Designs |
| 51 | (Case Reports.pt. or (case report? or case study or case studies).ti.) not (review* or trial*).ti,ab,kf,hw. | 2105067 |  |
| 52 | comment/ or editorial/ or (comment or editorial or news or newspaper article).pt. | 1634199 |  |
| 53 | (Case Reports.pt. or (case report? or case study or case studies).ti.) not (review* or trial*).ti,ab,kf,hw. | 2105067 |  |
| 54 | comment/ or editorial/ or (comment or editorial or news or newspaper article).pt. | 1634199 |  |
| 55 | (exp Animals/ or Models, Animal/ or Disease Models, Animal/) not Humans/ | 5071031 |  |
| 56 | ((animal or animals or canine* or dog or dogs or feline or hamster* or lamb or lambs or mice or monkey or monkeys or mouse or murine or pig or pigs or piglet* or porcine or primate* or rabbit* or rats or rat or rodent* or sheep* or veterinar*) not (human* or patient*)).mp. | 4937037 |  |
| 57 | 50 not (or/51-56) | 2476 |  |
| 58 | remove duplicates from 57 | 2471 |  |
